# Toward a Country-Based Prediction Model of COVID-19 Infections and Deaths Between Disease Apex and End: Evidence From Countries With Contained Numbers of COVID-19

**DOI:** 10.3389/fmed.2021.585115

**Published:** 2021-06-10

**Authors:** Tianshu Gu, Lishi Wang, Ning Xie, Xia Meng, Zhijun Li, Arnold Postlethwaite, Lotfi Aleya, Scott C. Howard, Weikuan Gu, Yongjun Wang

**Affiliations:** ^1^College of Graduate Health Science, University of Tennessee Health Science Center, Memphis, TN, United States; ^2^Department of Neurology, Beijing Tiantan Hospital, Capital Medical University, Beijing, China; ^3^Department of Basic Medicine, Inner Mongolia Medical University, Inner Mongolia, China; ^4^Department of Orthopedic Surgery and BME-Campbell Clinic, University of Tennessee Health Science Center, Memphis, TN, United States; ^5^College of Business, University of Louisville, Louisville, KY, United States; ^6^Department of Medicine, University of Tennessee Health Science Center, Memphis, TN, United States; ^7^Chrono-Environnement Laboratory, UMR CNRS 6249, Bourgogne Franche-Comté University, Besançon Cedex, France; ^8^College of Nursing, University of Tennessee Health Science Center, Memphis, TN, United States; ^9^Research Service, Memphis VA Medical Center, Memphis, TN, United States

**Keywords:** coronavirus, COVID-19, mortality, pandemic, prediction, infectious disease, death

## Abstract

The complexity of COVID-19 and variations in control measures and containment efforts in different countries have caused difficulties in the prediction and modeling of the COVID-19 pandemic. We attempted to predict the scale of the latter half of the pandemic based on real data using the ratio between the early and latter halves from countries where the pandemic is largely over. We collected daily pandemic data from China, South Korea, and Switzerland and subtracted the ratio of pandemic days before and after the disease apex day of COVID-19. We obtained the ratio of pandemic data and created multiple regression models for the relationship between before and after the apex day. We then tested our models using data from the first wave of the disease from 14 countries in Europe and the US. We then tested the models using data from these countries from the entire pandemic up to March 30, 2021. Results indicate that the actual number of cases from these countries during the first wave mostly fall in the predicted ranges of liniar regression, excepting Spain and Russia. Similarly, the actual deaths in these countries mostly fall into the range of predicted data. Using the accumulated data up to the day of apex and total accumulated data up to March 30, 2021, the data of case numbers in these countries are falling into the range of predicted data, except for data from Brazil. The actual number of deaths in all the countries are at or below the predicted data. In conclusion, a linear regression model built with real data from countries or regions from early pandemics can predict pandemic scales of the countries where the pandemics occur late. Such a prediction with a high degree of accuracy provides valuable information for governments and the public.

## Introduction

Disease modeling and prediction are important but difficult because of the great variations among infectious diseases (1). Multiple models have been developed to predict the total number of infections and deaths from COVID-19. Examples include the model by the U.S. Center for Disease Control and Prevention (CDC) (https://www.cdc.gov/coronavirus/2019-ncov/covid-data/forecasting-us.html), by the Institute for Health Metrics and Evaluation (IHME) (http://www.healthdata.org/covid/updates), the model at University of Washington (2), and at the Johns Hopkins coronavirus resource center (https://coronavirus.jhu.edu/). These models are useful, but changes had to be made constantly on their predictions based on new developments of COVID-19 (3, 4). Therefore, using real data from countries nearing the end of the pandemic to build prediction models may be an effective way to predict infections and deaths in countries where the pandemic is still ongoing.

Real data to build the predictions models were from countries including China, South Korea, and Switzerland, in which the COVID-19 pandemic has largely been controlled and its apex has already passed. Although the country-based conditions and pandemic situations in each country are very different, we believe that a careful analysis of the situations in these countries will help with predictions for countries where the pandemic is still developing and endemic (5). Although in some countries, the prevalence and incidence rate of the 2019 novel coronavirus (COVID-19) has passed its apex, and countries such as Italy and the UK are partially lifting restrictions, the decision to return to normal is still based on the trajectory of the pandemic and the accurate prediction of the pandemic's nadir (6).

In this study we tested whether a country-based model using data from China, South Korea, and Switzerland is useful for predictions for other countries. We conducted a comprehensive analysis of these data to build the model and then used the model to make predictions about the countries where the pandemic is still currently prevalent.

## Methodological Approach

### Data Collection

Data for COVID-19 disease prevalence and mortality from cities and provinces in China and other countries were obtained from public websites (7). Data were collected on daily cumulative total number of patients, new cases, cumulative total deaths, and new deaths. Data from China were collected from the period beginning Jan 19, 2020 up to March 19, 2020, when the daily new domestic case fell to zero. Data from other countries begins with the date of the first report of the number of COVID-19 patients through May 10 for establishment of the predictive model. Data for the model testing were collected before and up to March 30, 2021 from https://www.worldometers.info/coronavirus/. The newly updated data from all countries were collected from WHO daily situation report on COVID-19 at https://www.who.int/emergencies/diseases/novel-coronavirus-2019/situation-reports.

### Characterization of the COVID-19 Pandemic

Data were uploaded into an Excel spreadsheet and characterized with different parameters. For the cities and provinces in China, patterns of COVID-19 were defined by the parameters as follows. The time of the beginning of the COVID-19 pandemic is defined as the day the first COVID-19 patient was reported. The end of the pandemic was defined as the 1st day of zero new patients reported that was followed by no new patients reported continuously for the next 14 days. The whole pandemic period is defined as the day of the first reported COVID-19 patient to the day of the end of the pandemic (8–11). For data from China and other countries, the weighted numbers of patients and deaths were also calculated in intervals of 3, 5, and 7 days for estimating the apex day of the pandemic.

### Relationship Between the Number of Patients at Apex Days, Death Ratio, and the Length of the Period From Apex to the End

One important statistic is the length of time from the disease apex day to the end of the disease pandemic (as defined by the metric described above). Once we obtained the parameters above, we calculated the days from the apex day to the end of the pandemic for cities and provinces in China. The days from the “first report” day and from the end day to the apex day, which is defined as the day with the largest number on the average of 3, 5, and 7 days, were then calculated. The relationship between the days from apex day to first report day, and days from apex day to end day, was characterized by regression modeling. For infected people during the COVID-19 pandemic period, we divided numbers of people into two categories: the infected numbers from the beginning of the pandemic to the apex day, and infected numbers for the remaining days until the end of the pandemic period. For the relationship between numbers of people infected in these two categories, we used four regression models: linear, exponential, logarithmic, and power models. Similarly, we also divided the death numbers into the same two categories as that for infections. The relationship between the two categories was analyzed with a similar approach.

### Prediction of Days to the End of the Pandemic, Number of Infections (Or Infected Patients), and Deaths in The First Wave and Entire Pandemic in Top Pandemic Countries

Based on the mathematical models, we estimated the days from apex day to the end of the pandemic period, and the predicted future infection and death rates after the apex day in the 10 countries. These 10 countries are believed to be the countries with the highest prevalence of the epidemic with relatively reliable data on COVID-19. Multiple models were tested for the initial estimation, followed by estimations for the least and largest numbers for each of the three types of pandemic features.

The prediction made using data before May 10, 2020 was compared to predictions based on real data in the first wave of the pandemic and entire pandemic. The numbers of patients predicted based on the ratio before and after apex day and based on regression models are compared to the real numbers of patients at the end of the first pandemic and on the day of March 30, in the 14 countries. Similar comparisons were conducted for numbers of deaths.

### Statistical Analyses

For the correlational analysis, we followed our previous criteria: a significant correlation was defined as an *R* value equal to or more than 0.7 or −0.7 for either a strong positive or negative correlation, an *R* value between 0.35 and 0.69 or −0.35 and −0.69 was considered a moderate correlation, and an *R* value between 0 and 0.35 or 0 and −0.35 was considered as no correlation between the two measures. To build models for estimating the total number of patients and total mortalities based on prevalence up to the apex day, we compared the models by testing all multiple regression models, including linear, polynomial, logistic regression, and linear with power analysis. The best fit to the distribution of real data on the plots was selected as the estimation model.

## Results

### Characterizations of Apex Days of the COVID-19 Disease

**Figure 1:**
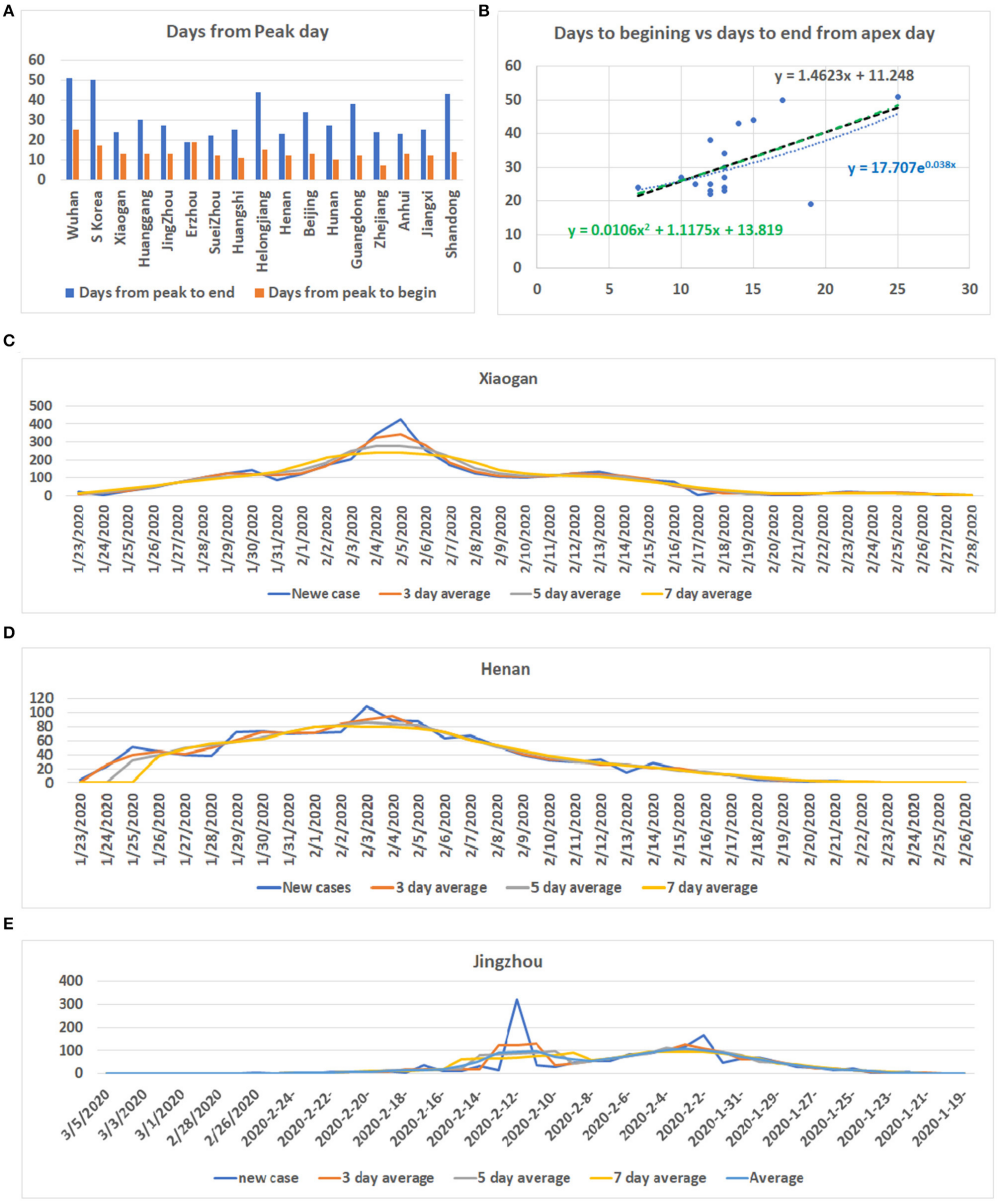
Information for COVID-19 pandemic. **(A)** The number of days from the beginning of the COVID-19 onset to the apex of onset and the number of days from the apex of onset to no new cases in each region. Vertical axis: days, Horizontal axis: cases. **(B)** Correlation between the date before the apex of onset and the date after the apex. Vertical axis: days after apex day, Horizontal axis: cases before and including apex day. **(C)** The distribution of the number of cases in Xiaogan City during all periods measured. It shows that there is a very obvious apex. **(D)** The daily distribution of patients in Henan Province during the pandemic. The apex onset date is not obvious. **(E)** The distribution of patients in Jingzhou shows that there are two apexes of incidence.

By analyzing data from nine provinces other than Hubei, the major cities of Hubei Province, and from South Korea and Switzerland, we determined the apex incidence in each region. Then we counted the date from the beginning of the disease epidemic to the apex (hereafter referred to as before apex day) and the date from the apex to the end of the pandemic. Overall, the date from the apex to the end of the outbreak was almost twice that of the period from the beginning to the apex of the disease (see Table 1). The average time from the day of first case reported to the apex day was 14 days, while the time between the apex day to the day of no new patients was more than 32 days. The ratio of before and after the apex day is 1:2.24. The correlation coefficient between these two time periods was 0.56 (see Table 1). Figure 1A shows the pre- and post-onset dates in each region. Figure 1B shows the correlation between the early stage and the late stage. Their relationship is expressed in the form of three models using linear, polynomial, and exponential regressions. At the same time, we noticed that the apex period of presentation is not the same everywhere (see Supplementary Figure 1). Some areas have an obvious apex period (see Figure 1C), while in some areas the apex period is relatively flat and not as obvious (see Figure 1D). What is more interesting is that some areas have a small apex period after the apical apex period (see Figure 1E).

**Table 1:** Information from pandemics in different cities, provinces, and countries for model construction.

**City**	**Days from apex day to end**	**Days from apex day to begin**	**No. of patients on peak day**	**Total patients**	**Total death**	**Death to apex day**
Wuhan	51	25	46,904	50,333	3,869	1,444
S Korea	50	17	4,335	10,683	237	32
Xiaogan	24	13	287	3,518	129	25
Huanggang	30	13	211	2,907	125	17
JingZhou	27	13	613	1,580	52	7
Erzhou	19	19	861	1,394	59	28
SueiZhou	22	12	706	1,307	45	8
Huangshi	25	11	509	1,015	39	2
Helongjiang	44	15	35	484	13	3
Henan	23	12	86	1,273	22	2
Beijing	34	13	24	485	8	1
Hunan	27	10	65	1,018	4	0
Guangdong	38	12	95	1,391	8	0
Zhejiang	24	7	98	1,234	1	0
Anhui	23	13	63	990	6	0
Jiangxi	25	12	65	935	1	0
Shandong	43	14	33	762	7	0
Switzerland	50	27	7,474	30,344	1,854	98
Summary	579	258	62,465	111,653	6,479	1,667
Average	32	14	3,470	6,203	360	93
Ratio		2.24		1.79		3.89
R	0.56		0.99		0.92	
R (W/o Wuhan)	0.56		0.97		0.94	

### Relationship of Infection Rates Before and After Apex Day

**Figure 2:**
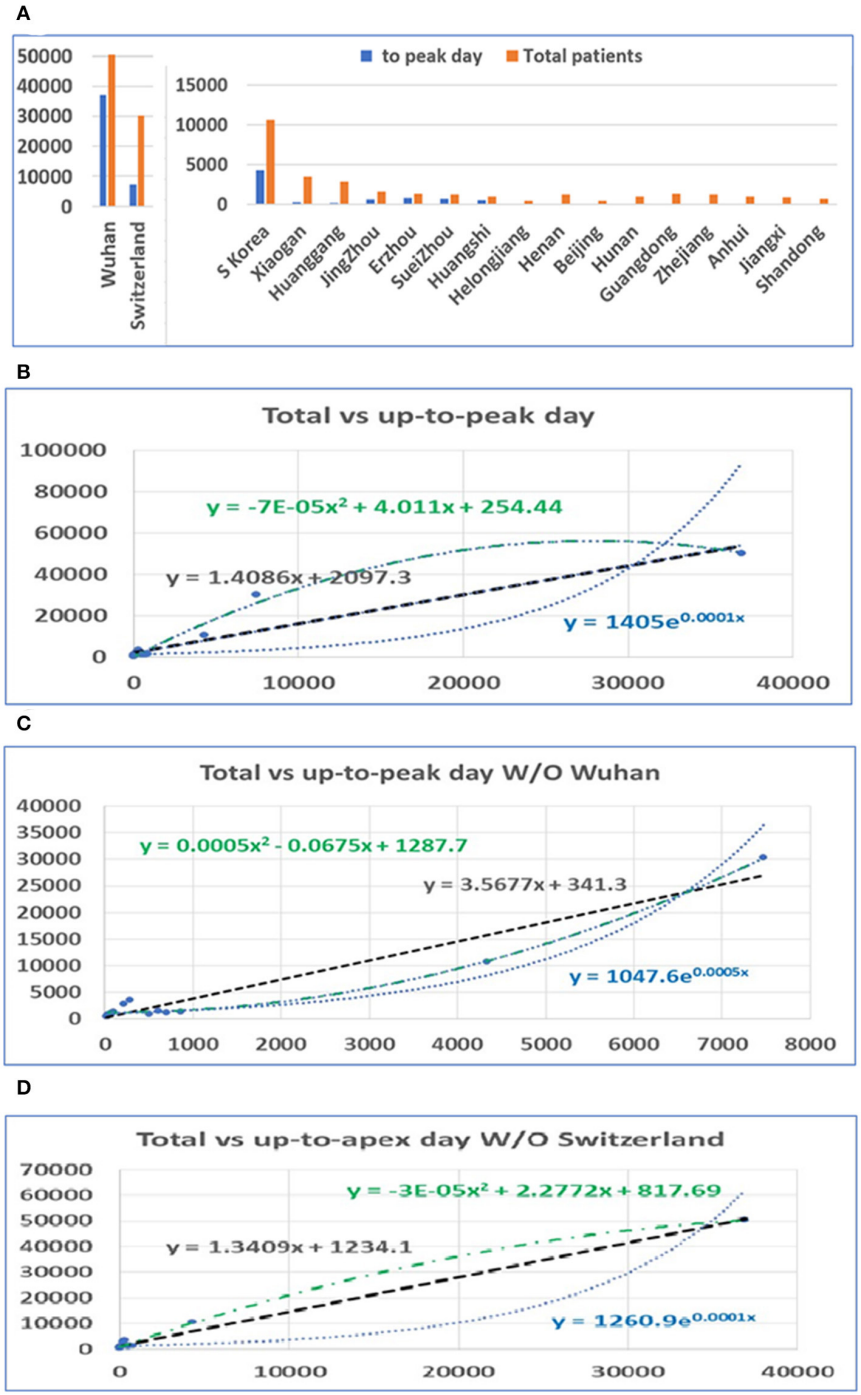
The distribution of patients before and after the pandemic apex. **(A)** The number of patients in major cities in Hubei Province in China, the provinces with the highest incidence, those in Switzerland, and those in South Korea before and after the apex of the pandemic. **(B)** The relationship between the total number of patients and the number of patients before the apex of the disease. The number on the vertical axis is the total number of cases, and the horizontal axis is the number of cases before the apex of the pandemic. **(C)** The relationship between the total number of cases and the number of cases before the apex period, excluding data from Wuhan. **(D)** The relationship between the total number of cases and the number of cases before the apex period, excluding data from Switzerland.

After the apex period of the COVID-19 pandemic was determined, we conducted statistical analyses of the number of patients before and after the apex period. Interestingly, the difference between the number of patients before and after the apex period is not as great as the difference between the days for the pandemic period before and after the apex day (see Figure 2A). Our analysis shows that when the data from Wuhan are included, the number of infected persons in the latter period of the outbreak is smaller than the number of patients before the apex period of the infection. If we exclude the data from Wuhan, in the latter period of the pandemic, the number of infected patients is slightly bigger than the previous number. However, the number of patients before the apex day and the total number of patients showed a significant correlation, regardless of whether data for Wuhan are included or not, with r values of 0.99 and 0.97 (see Table 1), respectively. Figures 2B,C show the relationship between the total number of people affected and the number of patients before apex day (see Figure 2B) and without data from Wuhan (see Figure 2C). Figure 2D shows the relationship between the total number of people infected and the number of patients before apex day without the data from Switzerland. The total numbers of patients before and after the apex are 62,465 and 111,653, respectively, with a ratio of 1:1.79 (see Table 1). However, if the data of Wuhan is excluded, numbers of patients before and after the apex are 15,561 and 61,320, respectively, and the ratio is 1:3.94. The models for these relationships were interpreted with multiple models including linear, polynomial, logarithmic power, and exponential regressions (see Supplementary Figure 2).

### Relationship Between Death Numbers Before and After the Apex Day

**Figure 3:**
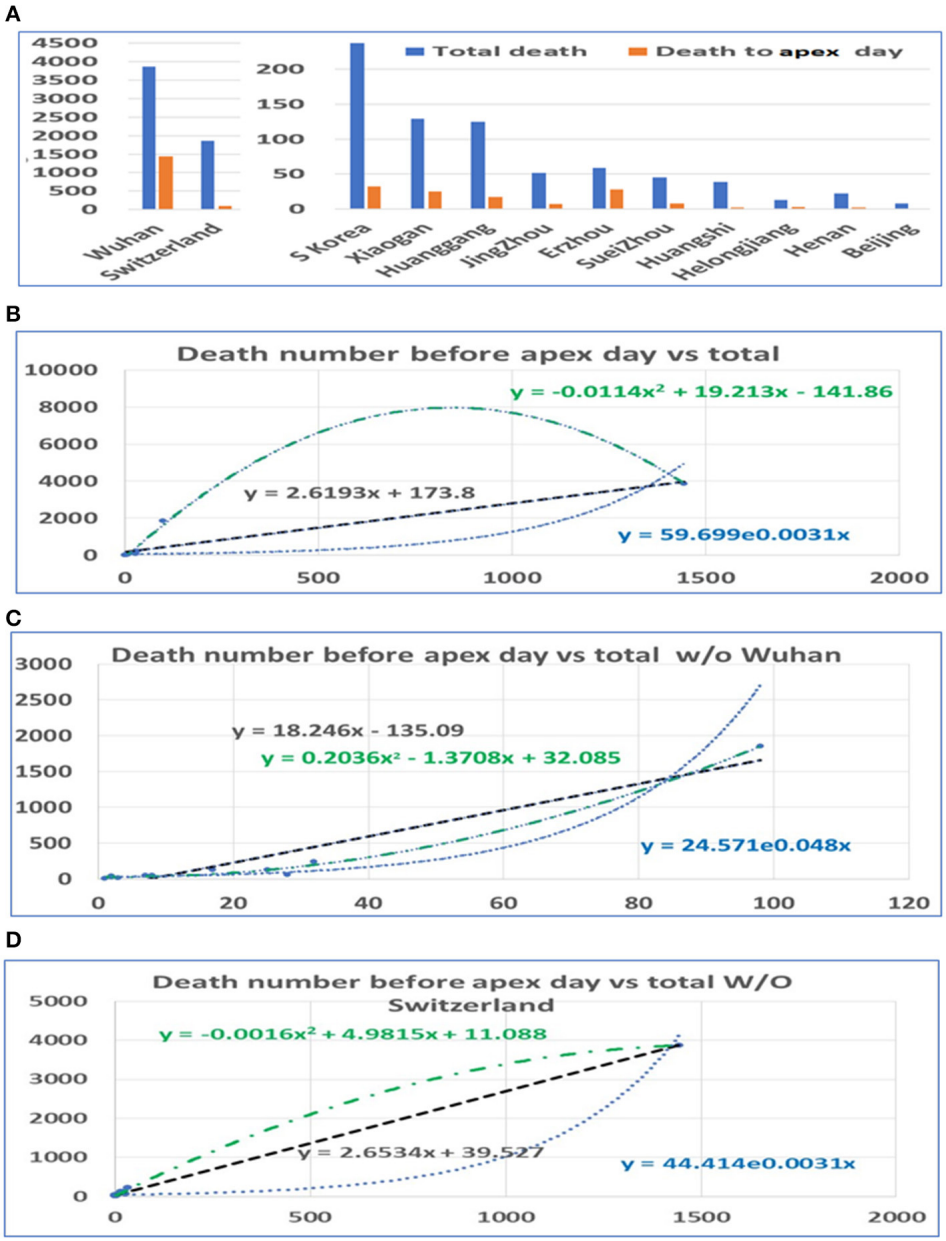
The distribution of mortality before and after the pandemic apex. **(A)** The mortality in major cities in Hubei Province in China, the provinces with the highest incidence, those in Switzerland, and those in South Korea before and after the apex of the pandemic. **(B)** The relationship between the total mortality and the mortality before the apex of the disease. The number on the vertical axis is the total mortality, and the horizontal axis is the mortality before the apex of the pandemic. **(C)** The relationship between the total mortality and the mortality before the apex period, excluding data from Wuhan. **(D)** The relationship between the total mortality and the mortality before the apex period, excluding data from Switzerland.

In a similar way, we conducted statistical analyses of the mortalities before and after the apex of the pandemic. The mortality rates before the apex day and the total for the pandemics among cities in China and among these three countries are highly correlated, with *r* = 0.92. Unlike the number of infections, the mortalities after the apex were several times higher than that before the apex. Similarly, we performed analyses both including and excluding data from Wuhan. The death toll in some provinces was zero before the apex. In order to prevent these provinces from being delayed for our statistics, we deleted the data from these regions in the analysis of the mortalities (see Figure 3A). Even so, the mortalities between the apex and the end of the outbreak are at least double the mortality before the apex. The correlation between the number of deaths before the apex day and number of total deaths is still high, with an *r* value of 0.92 with Wuhan, and 0.95 without Wuhan (see Table 1). The mortality numbers after the apex day are 3.87 and 11.58 times more than that before the apex day, with and without the data from Wuhan, respectively. Accordingly, the three types of regression models were performed between the death number before apex day and the total death numbers (see Figures 3B,C). Similar to the infected patient data, these models included linear, polynomial, and exponential regressions (see Supplementary Figure 3). The models in Figure 3B included the data from Wuhan while those in Figure 3C did not. The models in Figure 3D shows the relationship without the data from Switzerland. These results indicate that many infected people who did not die before the apex day may die within a few days or even a dozen days after the apex period.

### Prediction of Disease Extended Days, Infected Populations, and Deaths After the Apex Day of the COVID-19 Pandemic

Based on our estimation of the date of the pandemic before and after the apex of the COVID-19 pandemic, we predicted the future incidence of COVID-19 (beginning May 10, 2020) for several countries (see Supplementary Table 1). The data from some of the countries suggest that the disease has reached its apex (see Supplementary Figure 4). However, these data indicated that the predicted number of people infected in various countries after May 10 still ranges from at least 10,000 to tens of thousands. The maximum number of patients in the future for Japan, Iran, France, Italy, Spain, Germany, the UK, Netherland, Belgium, and the US are 5,220, 54,204, 349,134, 120,913, 142,082, 27,856, 88,157, 48,214, 51,619, and 340,713 based on simple ratio and 5,854, 57,405, 358,878, 127,308, 149,729, 31,722, 94,019, 50,109, 53,767, and 372,081 based on polynomial regression model, respectively.

**Figure 4:**
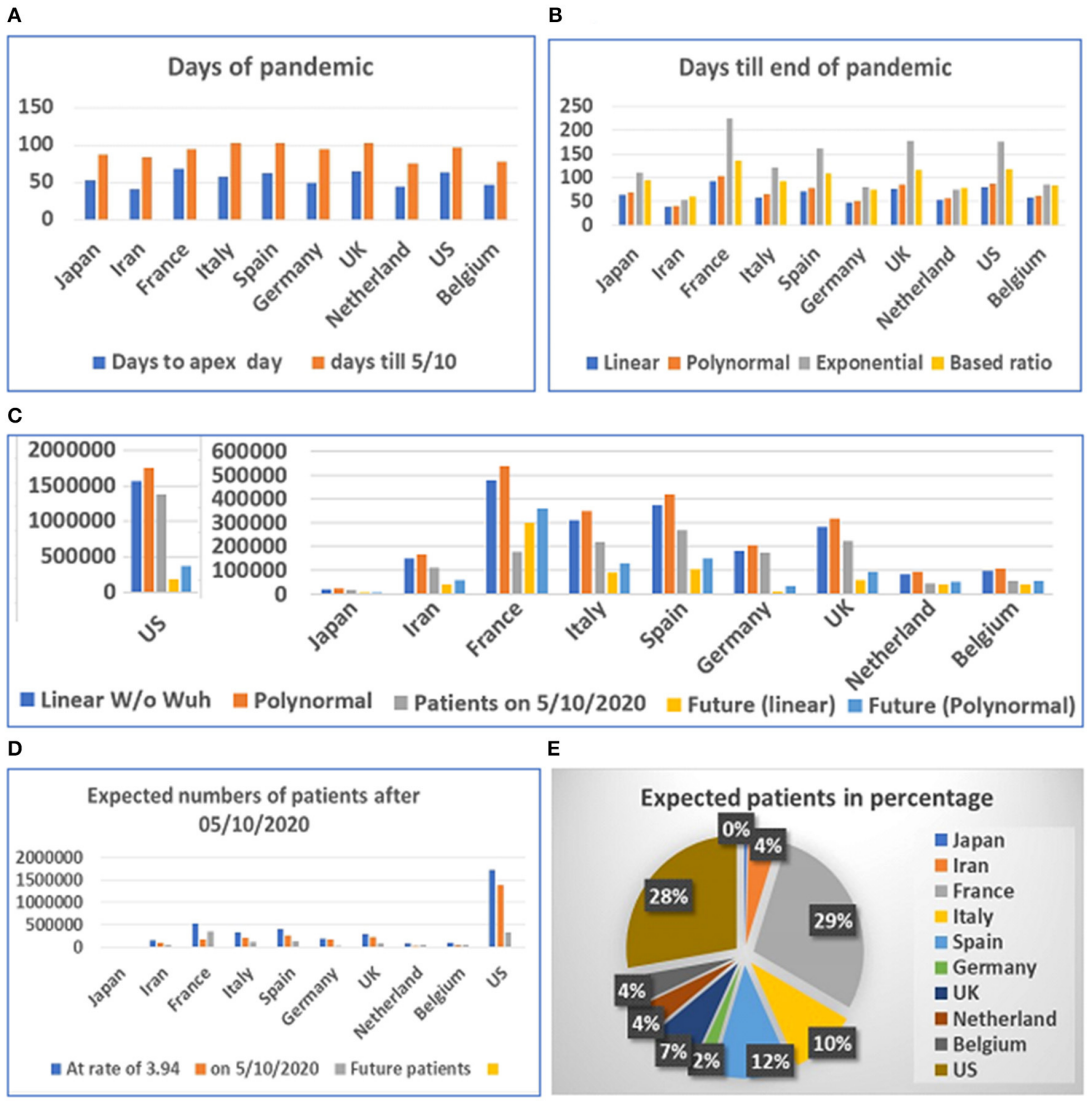
Days and number of patients predicted after May 1, 2020 in 10 countries. **(A)** Days of pandemic from beginning to apex day and to May 1, 2020. **(B)** Days remaining before end of pandemic predicted with different models. **(C)** Future numbers of patients after May 10, 2020, predicted by linear model (W/O data from Wuhan) and polynomal model. **(D)** Proportions of expected patients in different countries based on calculated ratio before and after apex day from data from China, Switzerland, and South Korea. **(E)** Proportion of expected patients in different countries.

The prediction of the length of the onset date after the apex period (see Figure 4A) was based on modeling by linear, polynomial, and exponential equations. Time was also calculated based on the ratio between days before and after the apex day. According to the results of these calculations, at least 3 weeks to 1 and 3 months are needed for the most affected countries to end the pandemic. The longest predicted pandemic durations are for France, the UK, and the US (see Figure 4B).

The prediction of the duration between the apex day and end day of disease was tested using multiple models. However, final predicted values were based on the linear and polynomial models (see Figure 4C). Our results suggest that the straight-linear equation produced negative values, while the data calculated by the exponential equation resulted in extraordinarily large numbers (see Supplementary Table 2). The model predicted that the number of patients in the US may exceed 1.7 million, while predicted patients in France may reach half a million before the end of the COVID-19 outbreak. We then calculated the number of patients before and after the apex day based on simple ratio, between before and after the apex day, which was found to be 1:3.94, calculated from data from China, South Korea, and Switzerland. The results predict that the US and France will have fewer than half a million more patients before the end of the pandemic (see Figures 4D,E).

### Test the Predictability of Models With Data of the Apex Day and Accumulated Data in the First Wave of the COVID-19 Pandemic

**Figure 5:**
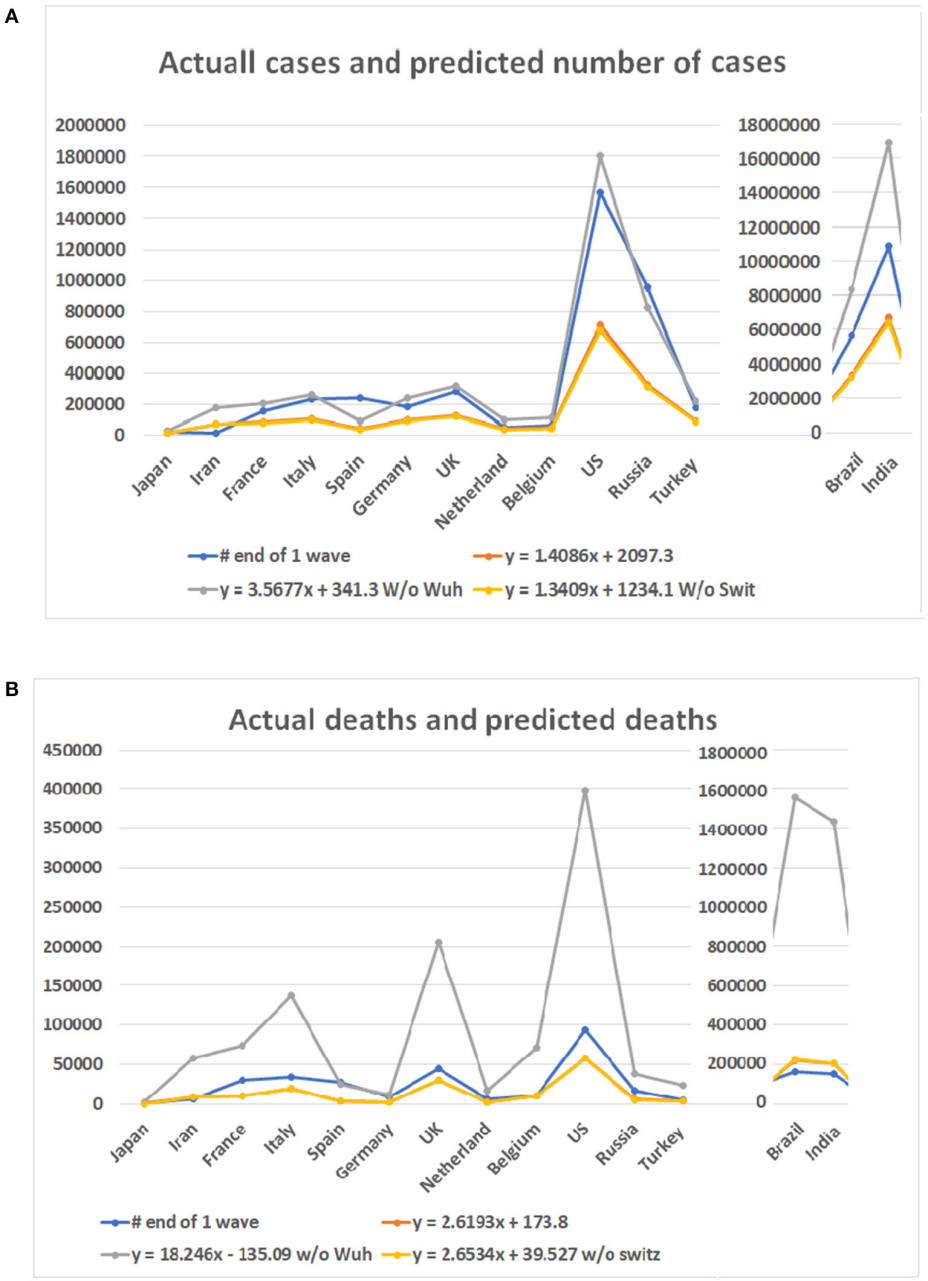
Case numbers and mortality predicted for 14 countries and real numbers in the first wave of the disease. Numbers on Y bar are the case of death numbers. Color code: Blue for the real numbers. Red for the predicted numbers based on linear model. Gray color for the predicted numbers based on the linear model without data from Wuhan. Point for predicted numbers without data of Switzerland. **(A)** Predicted number of cases based on three linear models and real number of cases. **(B)** Death numbers predicted based on the linear models and reported numbers from 14 countries.

Among the six mathematical models (see Figures 3B–D), only the linear models and one polynomial model converged. The first of the three linear models included the 10 locations while the second and third excluded data from Wuhan and Switzerland, respectively. The polynomial model excluded data from Wuhan. Other models produced either negative values or apparently aberrant data (see Supplementary Table 3). As the world failed to contain the COVID-19 disease, multiple waves of disease have occurred in many countries. We then compared the predicted case and death numbers from these three minor models and compared them to the real data in the first wave of the pandemic in 14 countries, which include the original 10 countries and four countries which were among the top 10 on the COVID-19 pandemic based on the case numbers (Figure 5). By comparing data from these three models, we obtained the minimum and maximum numbers of potential cases and deaths from each of these 14 countries (see Figures 5A,B). The maximum number of deaths in the future for Japan, Iran, France, Italy, Spain, Germany, the UK, Netherland, Belgium, the US, Brazil, India, Russia, and Turkey are 27,520, 180,395, 203,661, 265,728, 91,303, 238,967, 316,514, 95,067, 109,474, 1,805,451, 8,360,768, 16,962,457, 828,915, and 218,146, respectively. The minimum number of cases are 11,449, 68,907, 77,651, 100,978, 35,422, 90,920, 120,066, 36,836, 42,251, 679,675, 3,143,454, 6,376,350, 3,12,649, and 83,095, respectively. The actual number of cases in these countries are 16,851, 11,635, 156,156, 234,013, 242,707, 185,416, 285,420, 50,005, 60,550, 1,568,448, 5,631,181, 10,826,363, 956,749, and 175,218, respectively (Figure 5A). While data from most of the countries falls into the predicted range, the reported case from Iran is less than the predicted minimum number and Spain and Russia is more than the predicted maximum number of cases.

For the number of deaths, the maximum number predicted for these countries are 2,474, 57,522, 73,433, 136,765, 25,063, 11,634, 206,574, 17,454, 71,079, 398,175, 1,555,117, 1,433,745, 38,473, and 23,512, respectively. The actual numbers of deaths in all but Spain are less than the predicted maximum numbers. The minimum number predicted for these countries are 419, 8,424, 10,735, 19,826, 3,704, 1,751, 29,848, 2,597, 10,396, 57,353, 223,438, 206,014, 5,654, and 3,478, respectively. The death numbers of four countries, Iran, Belgium, Brazil, and India, are less than the predicted minimum numbers of deaths.

### Test the Predictability of Models With Data of the Apex Day and Total Accumulated Data of the Updated COVID-19 Pandemic Period

**Figure 6:**
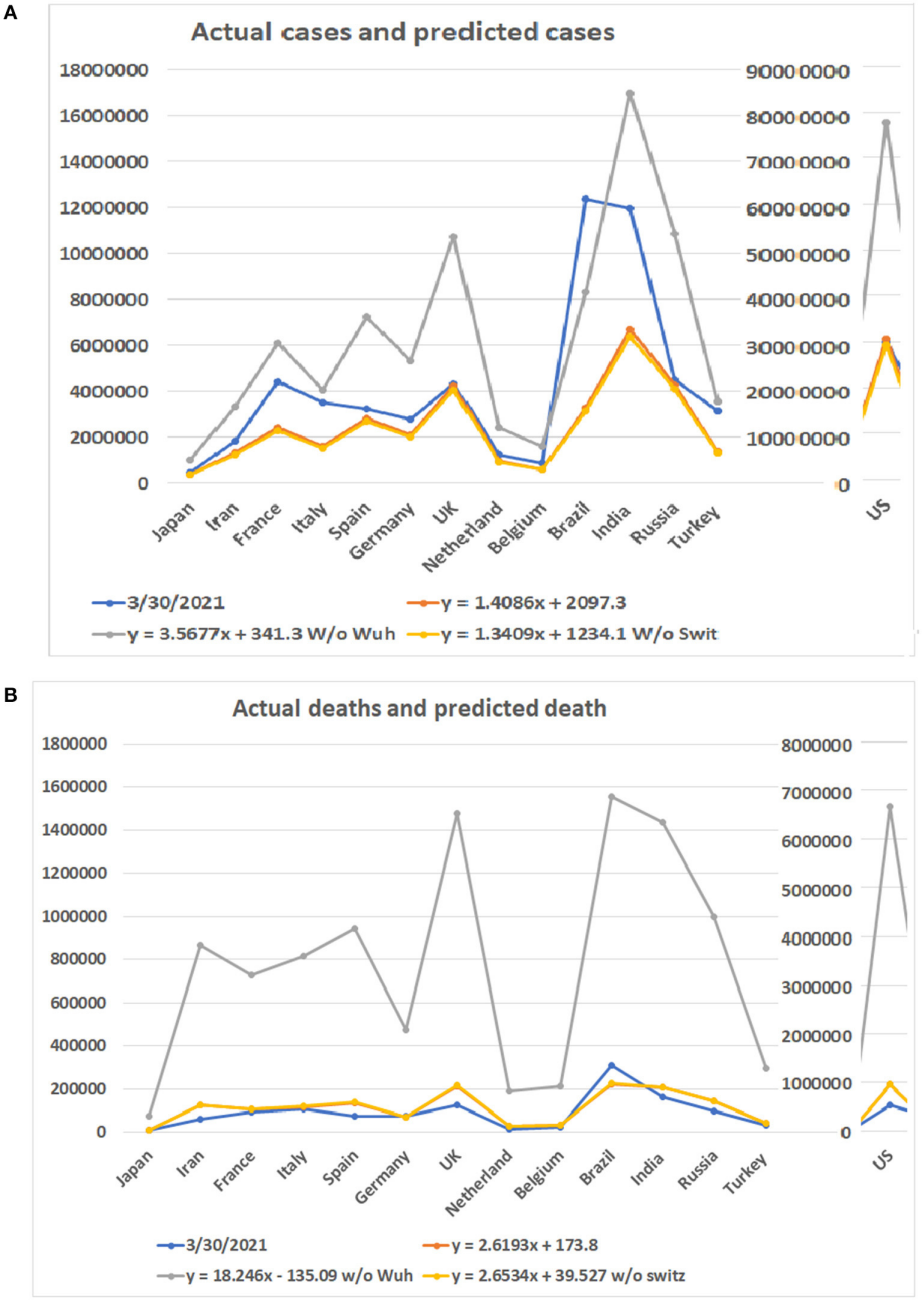
Comparison of predicted numbers of patients and deaths on March 30, 2021 to the real numbers. Numbers on Y bar are the case of death numbers. Color code: Blue for the real numbers up to March 30, 2021. Red for the predicted numbers based on linear model. Gray color for the predicted numbers based on the linear model without data from Wuhan. Point for predicted numbers without data from Switzerland. **(A)** Predicted number of cases based on three linear models and real number of cases. **(B)** Predicted number of deaths based on three linear models and real number of deaths.

As the COVID-19 pandemic continues to spread worldwide, we tested our models with updated data up to March 30, 2021. The highest apex among multiple waves was used as the apex of the entire pandemic of a country. The accumulated case and death numbers before the apex were used to predict the total number of cases and deaths. The predicted numbers than were compared to the accumulated total number of cases and deaths up to March 30, 2021. Figure 6 shows the predicted minimum and maximum numbers of patients and deaths based on the linear models and the real numbers on March 30, 2021. The total maximum numbers of cases for these 14 countries are 1,002,062, 3,338,991, 6,116,667, 4,083,759, 7,226,932, 5,330,517, 10,765,566, 2,414,208, 1,590,804, 77,637,725, 8,360,768, 16,962,457, 10,882,711, and 3,551,883. The predicted minimum number of cases for these countries are 377,725, 1,256,047, 2,300,021, 1,535,964, 2,717,308, 2,004,551, 4,047,283, 908,472, 599,001, 29,180,808, 3,143,454, 6,376,350, 4,091,312, and 1,336,061. A surprising finding is that the number of patients in Brazil has surpassed predicted maximum numbers by all three models (Supplementary Table 4). For the rest of the 13 countries, none of the numbers of patients on March 30, 2021 have surpassed the predicted maximum numbers of patients. Notably, the patient numbers of all countries have surpassed the minimum numbers of patients predicted by the regression model (see Figure 6A).

Regarding the number of deaths, the actual numbers of deaths in 12 out of 14 countries are less than the predicted minimum numbers of the deaths (Figure 6B). Only the death numbers of two countries, Germany and Brazil, are more than the predicted minimum numbers of deaths but have not reached the maximum numbers. The predicted minimum numbers of deaths in the future for Japan, Iran, France, Italy, Spain, the UK, Netherland, Belgium, the US, India, Russia, and Turkey are 1,610, 62,246, 10,666, 9,576, 61,145, 85,418, 11,106, 8,012, 415,536, 44,462, 45,914, and 11,681 (Supplementary Table 5).

## Discussion

Our data shows that real-time models and predictions are relatively reliable. The models may be utilized in the future for the case and death number prediction of pandemics of similar diseases. Due to the influence of societal factors and policies of different countries and regions, experience, or previous evidence-based models have considerable limitations in the predictions and estimations of the current COVID-19 pandemic. We tested a systematic prediction algorithm of the development of the COVID-19 disease from the pre-onset period to the turning point or apex day to after the turning point through analysis of real data from mainland China, South Korea, and Switzerland. Our analysis showed that the second half of the pandemic has more infections and deaths than the first half of the pandemic. This result suggests that the disaster caused by the COVID-19 pandemic is far from over. Unfortunately, the multiple waves of the pandemic in many countries around the world indicates such a prediction is true.

The linear model is used to predict the cases and deaths most close to the real numbers. However, we now realized that when the number of cases in a country or a region is below a single digit number for a consecutive 12 days, it indicates that a wave of the pandemic is ending but it is not necessarily the end of the whole pandemic. Therefore, we tested the models with the data from the first wave and the data of the entire pandemic period up to March 30, 2021. In both cases, the real data either fall in or close to the predicted data.

While most of the real data agrees with data predicted from the models, there are exceptions. In the test for the first wave of the disease, the reported case from Iran is less than the predicted minimum number, and Spain and Russia it is more than the predicted maximum number of cases. In the prediction of death numbers, for the first wave, the death numbers of Iran, Belgium, Brazil, and India are less than the predicted minimum numbers of deaths. The death numbers from these countries for the entire pandemic period are less than the predicted. This result is expected because the pandemic period has not yet ended, and the death apex usually comes later than the apex of case numbers. Thus, more deaths are expected before the end of the pandemic.

It is also important to note that our estimations are based on conditions of lockdowns in major cities, maintaining social distance, and wearing personal protective equipment (PPE). If the protective measures are not maintained in the later stages of the pandemic, the number of patients and deaths will be more than those predicted in our analyses. The pandemic was not ended as predicted.

Multiple social and environmental factors can influence the case and death numbers (12–15). At present, the differences between the infection rate and lethality of the 2019-nCoV among different populations are not clear. There has been no systematic analysis of any variation between infection rates and lethality of different mutations of the 2019-nCoV among different populations. In addition, we cannot rule out the possibility that different environmental and societal conditions may have an impact on viral infection rates and mortality rates. In particular, the medical system and availability of treatment methods for a disease directly affect the death rate of any infectious disease.

Initial knowledge of any novel disease is inherently limited (16–18). The data from Wuhan have been revised since the initial outbreak. Therefore, in our statistics, we evaluated pandemic data both with and without data from Wuhan. The prediction without Wuhan's data seems more accurate.

## Conclusion

A linear regression model built with real data from early COVID-19 pandemics can predict pandemic scales of later disease waves. Such a prediction with a high degree of accuracy benefits disease control and provides valuable information for governments and the public. However, many factors may influence the predictivity of the model. The model may need to be modified based on different situations.

## Data Availability Statement

The original contributions presented in the study are included in the article/Supplementary Material, further inquiries can be directed to the corresponding author/s.

## Author Contributions

TG, LW, WG, XM, LA, AP, SH, and YW: conceived and designed the experiments. TG, LW, NX, and WG: performed data searching, collection, and analyzed the data. XM, YW, ZL, WG, and AP: contributed analysis tools. TG, LW, NX, XM, ZL, AP, LA, SH, WG, and YW: wrote the manuscript. All authors: revised and approved the manuscript.

## Conflict of Interest

The authors declare that the research was conducted in the absence of any commercial or financial relationships that could be construed as a potential conflict of interest.

## Supplementary Material

Supplementary Figure 1 Disease infection apex and relations of different regions.

Supplementary Figure 2 Relationship between number of infected persons before the apex day and the total number.

Supplementary Figure 3 Death numbers and relations before apex day and total.

Supplementary Figure 4 Patterns of numbers of infected persons and peak day of investigated countries.

Supplementary Table 1 Days before and after apex day.

Supplementary Table 2 Calculation of the numbers of patients from the first wave.

Supplementary Table 3 Calculation of the numbers of deaths from the first wave.

Supplementary Table 4 Calculation of the numbers of patients from the entire pandemic.

Supplementary Table 5 Calculation of the numbers of deaths from the entire pandemic.
