# Supplementary material for: Toward a Country-Based Prediction Model of COVID-19 Infections and Deaths Between Disease Apex and End: Evidence From Countries With Contained Numbers of COVID-19
Source: Front Med (Lausanne). 2021 Jun 10;8:585115. doi: 10.3389/fmed.2021.585115 (PMC8222531; doi:10.3389/fmed.2021.585115)
Supplement: Supplementary Figure 1 — Disease infection apex and relations of different regions. [file Data_Sheet_1.PDF]

Supplemental Figure 1A. Relation between the days from beginning to the peak day and from peak day to the end of epidemic.

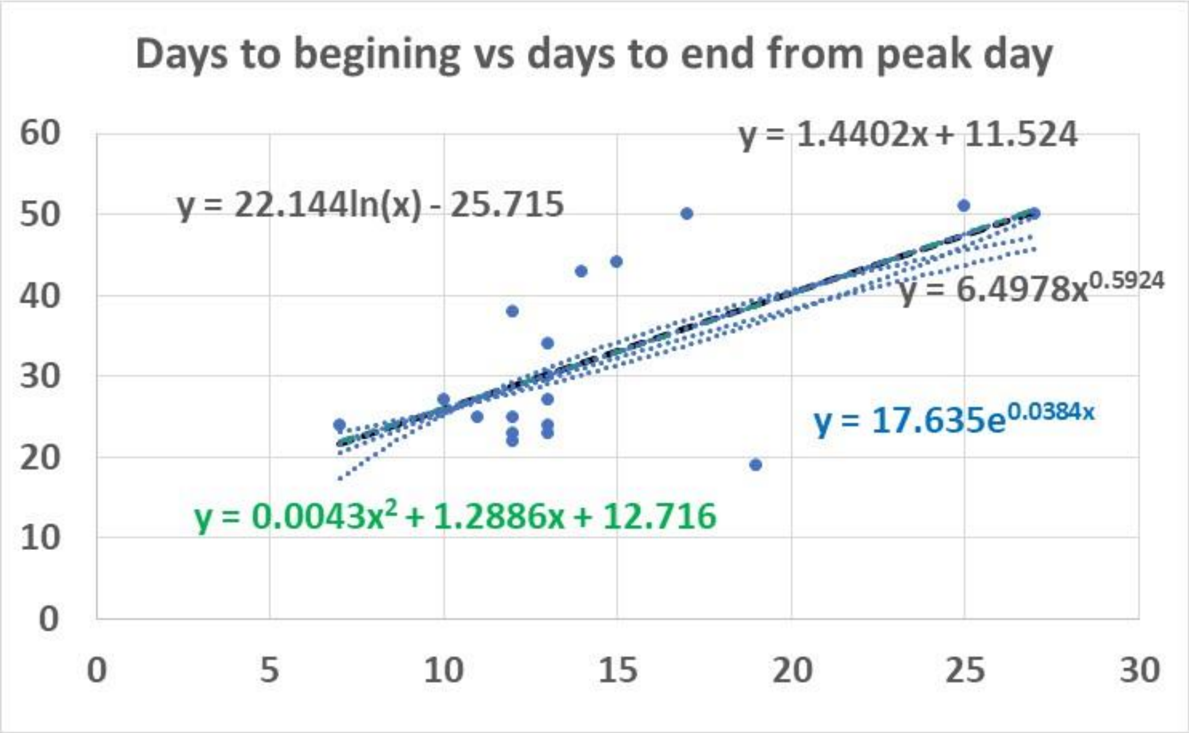

Supplemental Figure 1B. Distribution of number of patients in Wuhan city.

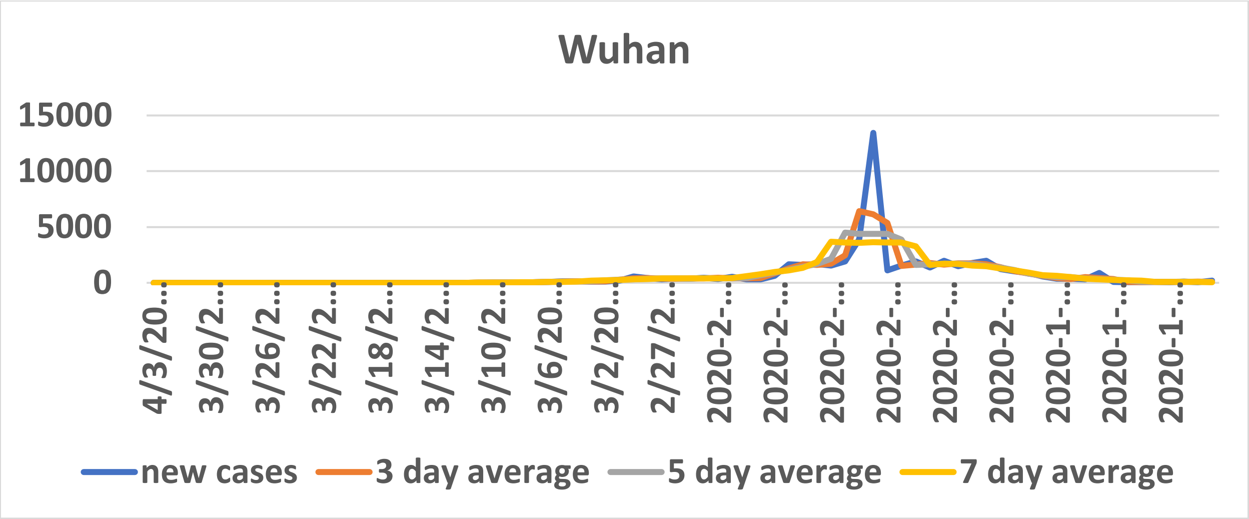

Supplemental Figure 1C. Distribution of number of patients in Helongjinag province.

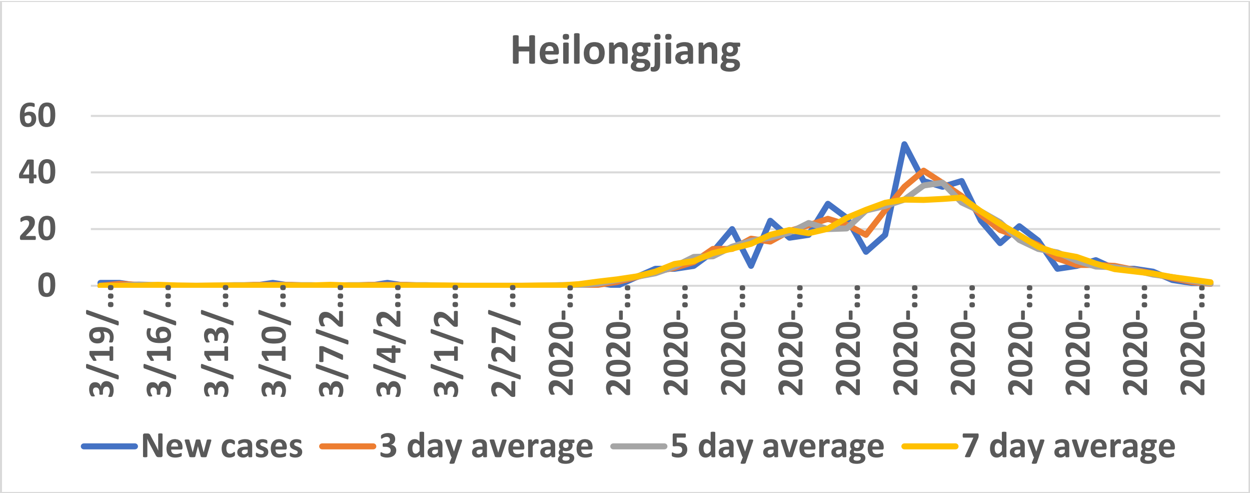

Supplemental Figure 1D. Distribution of number of patients Huan province.

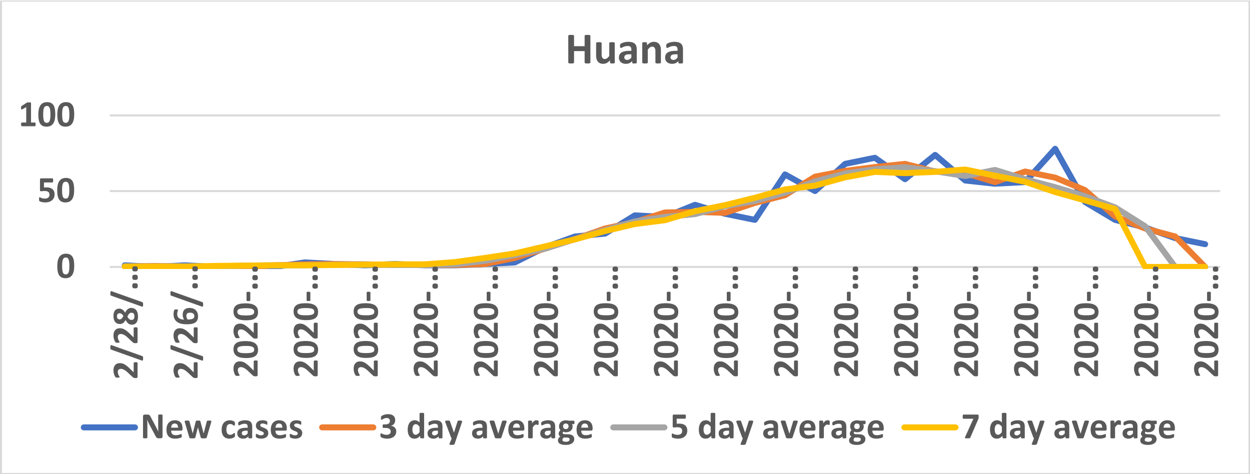

Supplemental Figure 1E. Distribution of number of patients Guangdong province.

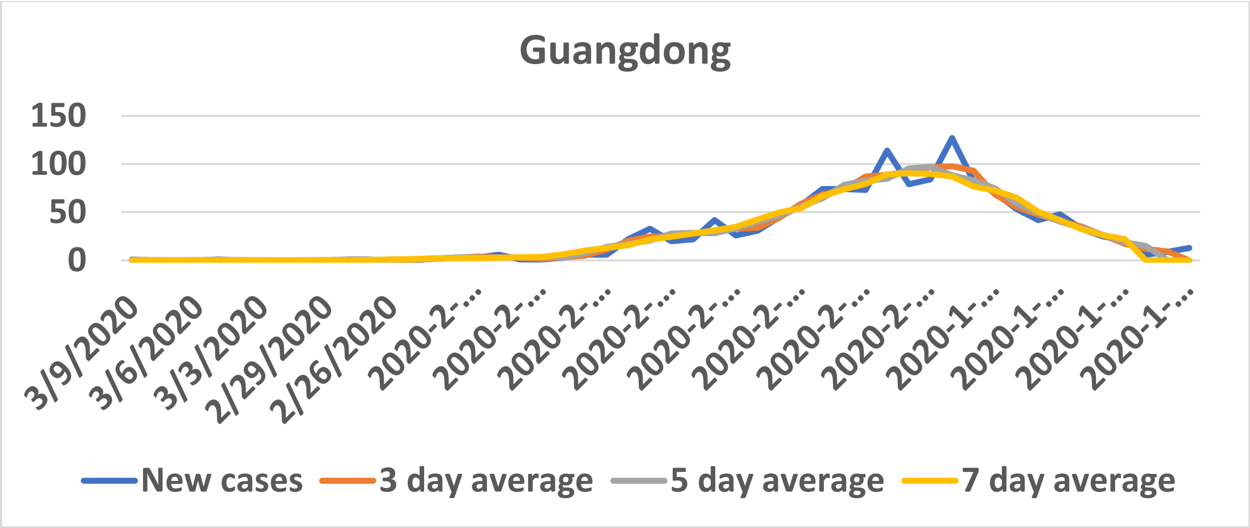

Supplemental Figure 1F. Distribution of number of patients Zhejiang province.

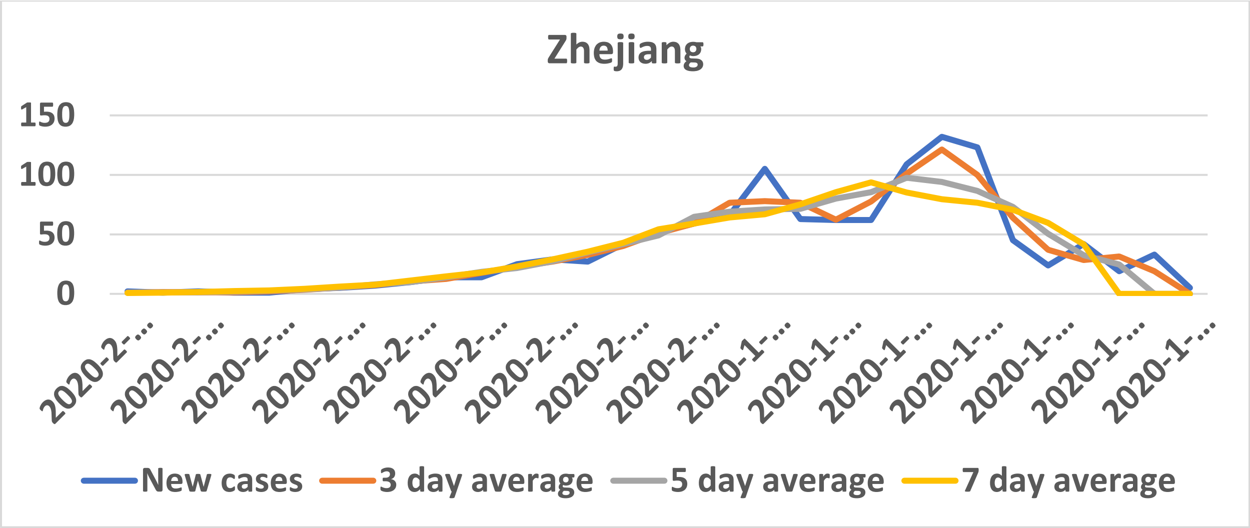

Supplemental Figure 1G. Distribution of number of patients Anhui province.

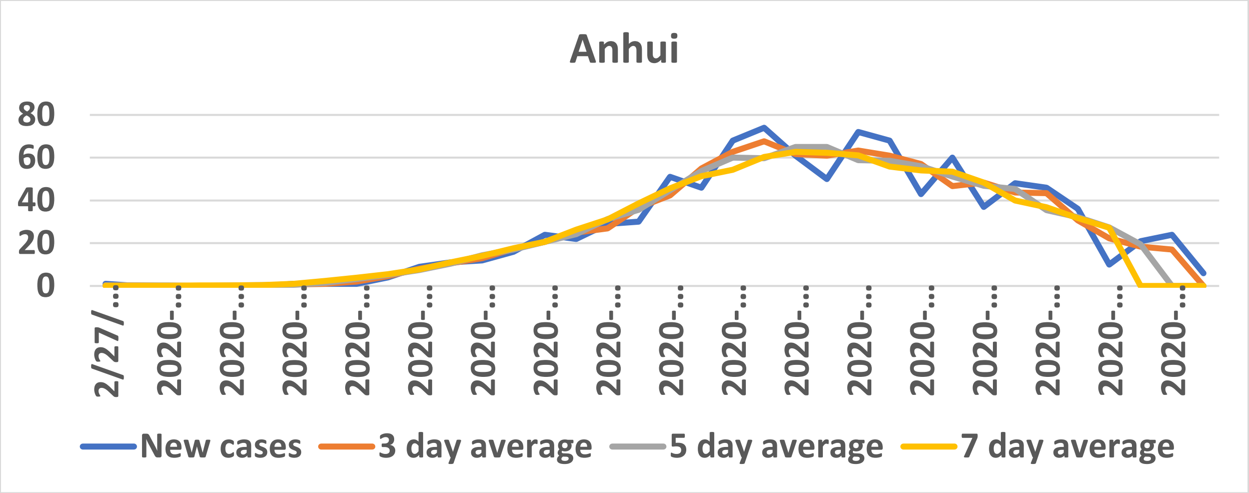

Supplemental Figure 1H. Distribution of number of patients Jiangxi province.

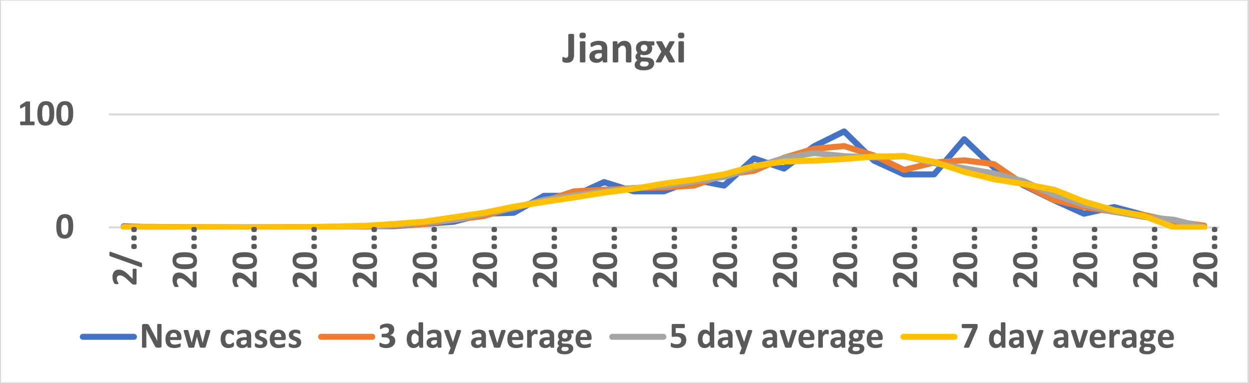

Supplemental Figure 1I. Distribution of number of patients Beijing City.

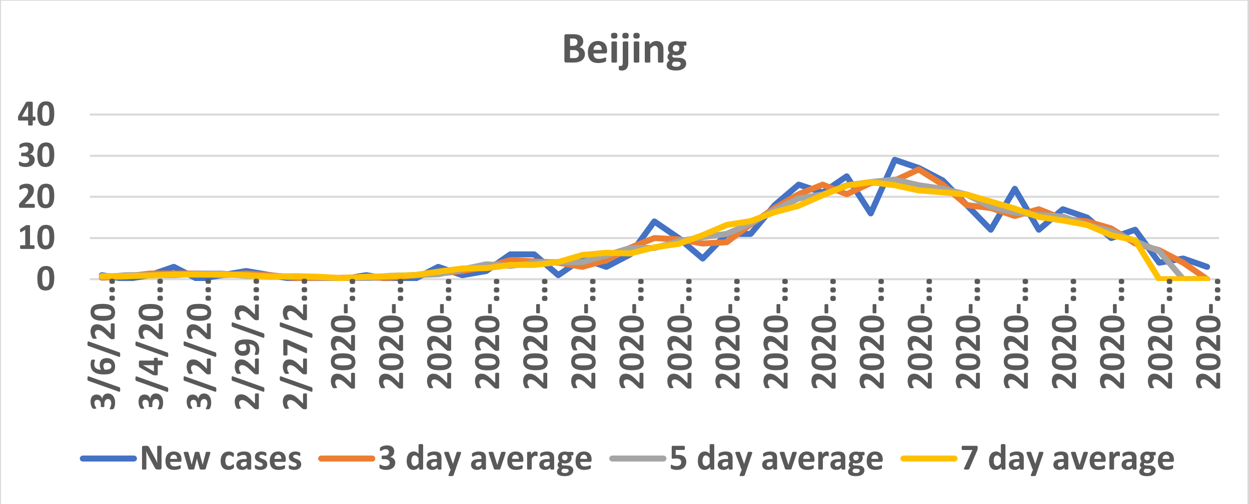

Supplemental Figure 1J. Distribution of number of patients Shandong province.

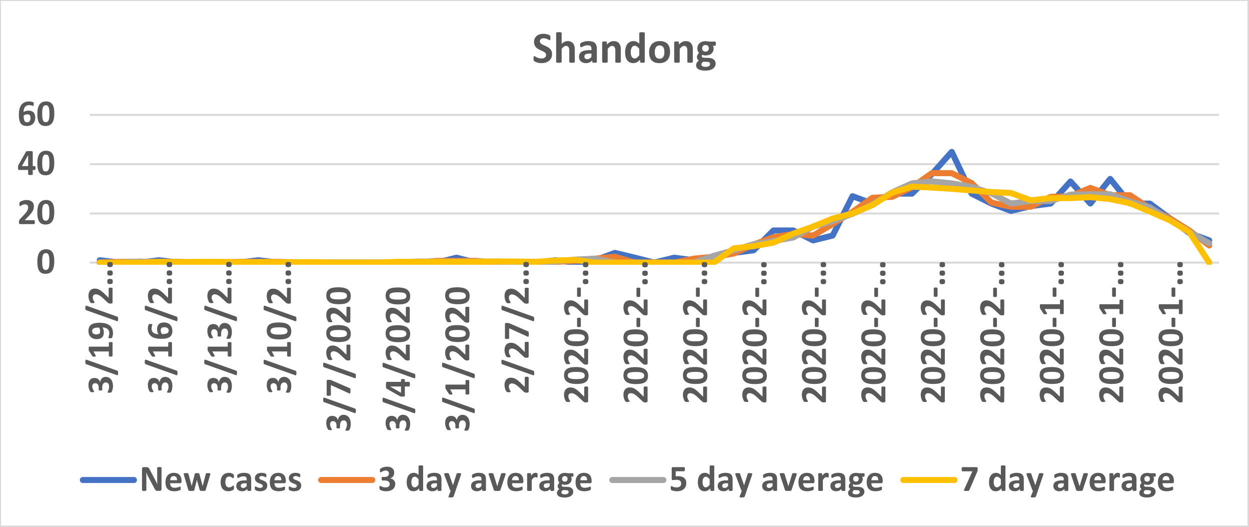
Shandong
60
40
20
0
3/19/2...
3/16/2...
3/13/2...
3/10/2...
3/7/2020
3/4/2020
3/1/2020
2/27/2...
2020-2-...
2020-2-...
2020-2-...
2020-2-...
2020-2-...
2020-2-...
2020-2-...
2020-2-...
2020-1-...
2020-1-...
2020-1-...
New cases
3 day average
5 day average
7 day average

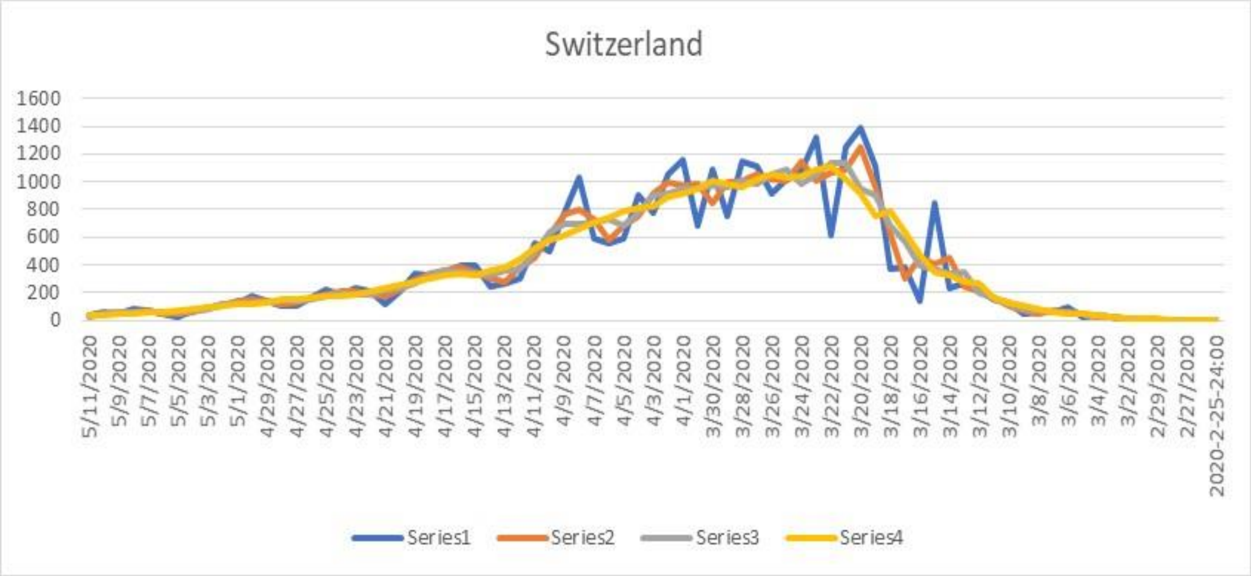
Switzerland
1600
1400
1200
1000
800
600
400
200
0
5/11/2020
5/9/2020
5/7/2020
5/5/2020
5/3/2020
5/1/2020
4/29/2020
4/27/2020
4/25/2020
4/23/2020
4/21/2020
4/19/2020
4/17/2020
4/15/2020
4/13/2020
4/11/2020
4/9/2020
4/7/2020
4/5/2020
4/3/2020
4/1/2020
3/30/2020
3/28/2020
3/26/2020
3/24/2020
3/22/2020
3/20/2020
3/18/2020
3/16/2020
3/14/2020
3/12/2020
3/10/2020
3/8/2020
3/6/2020
3/4/2020
3/2/2020
2/29/2020
2/27/2020
2020-2-25-24:00
Series1
Series2
Series3
Series4
