# Supplementary material for: Toward a Country-Based Prediction Model of COVID-19 Infections and Deaths Between Disease Apex and End: Evidence From Countries With Contained Numbers of COVID-19
Source: Front Med (Lausanne). 2021 Jun 10;8:585115. doi: 10.3389/fmed.2021.585115 (PMC8222531; doi:10.3389/fmed.2021.585115)
Supplement: Supplementary Figure 2 — Relationship between number of infected persons before the apex day and the total number. [file Data_Sheet_2.PDF]

Supplemental Figure 2. Total number of patients vs patients up to apex day.

Supplemental Figure 2A.

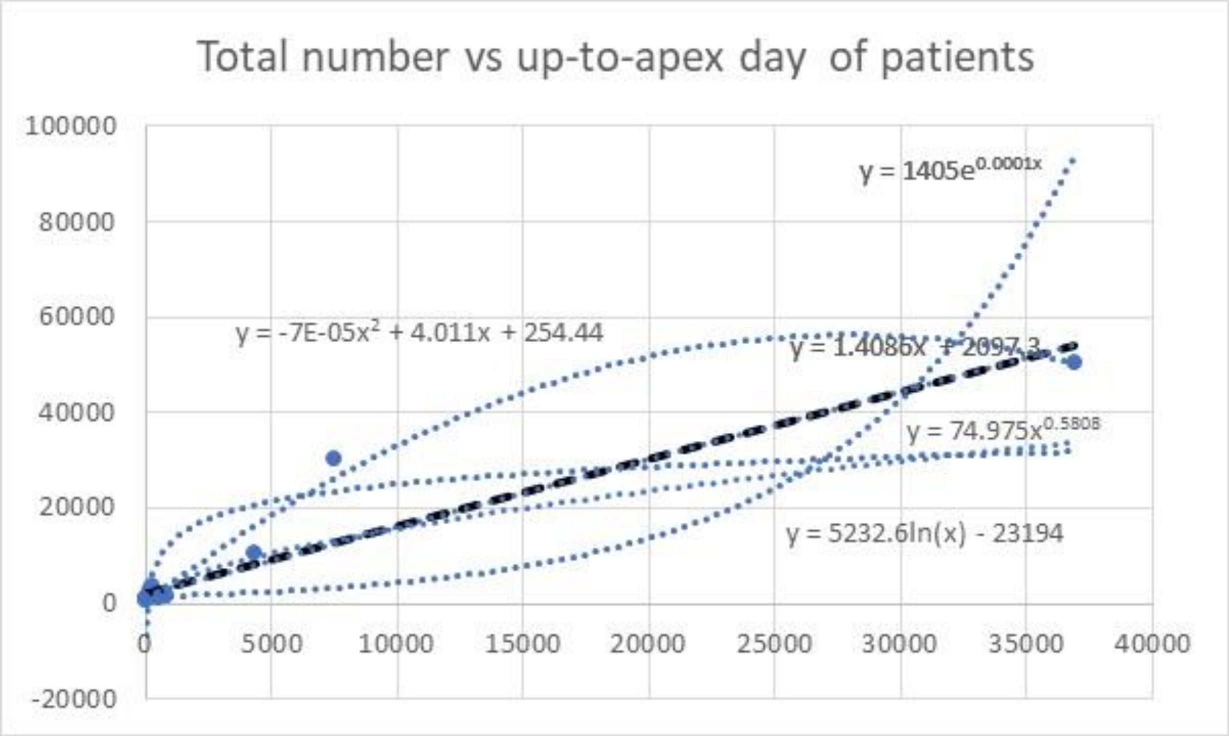

Supplemental Figure 2**B.**

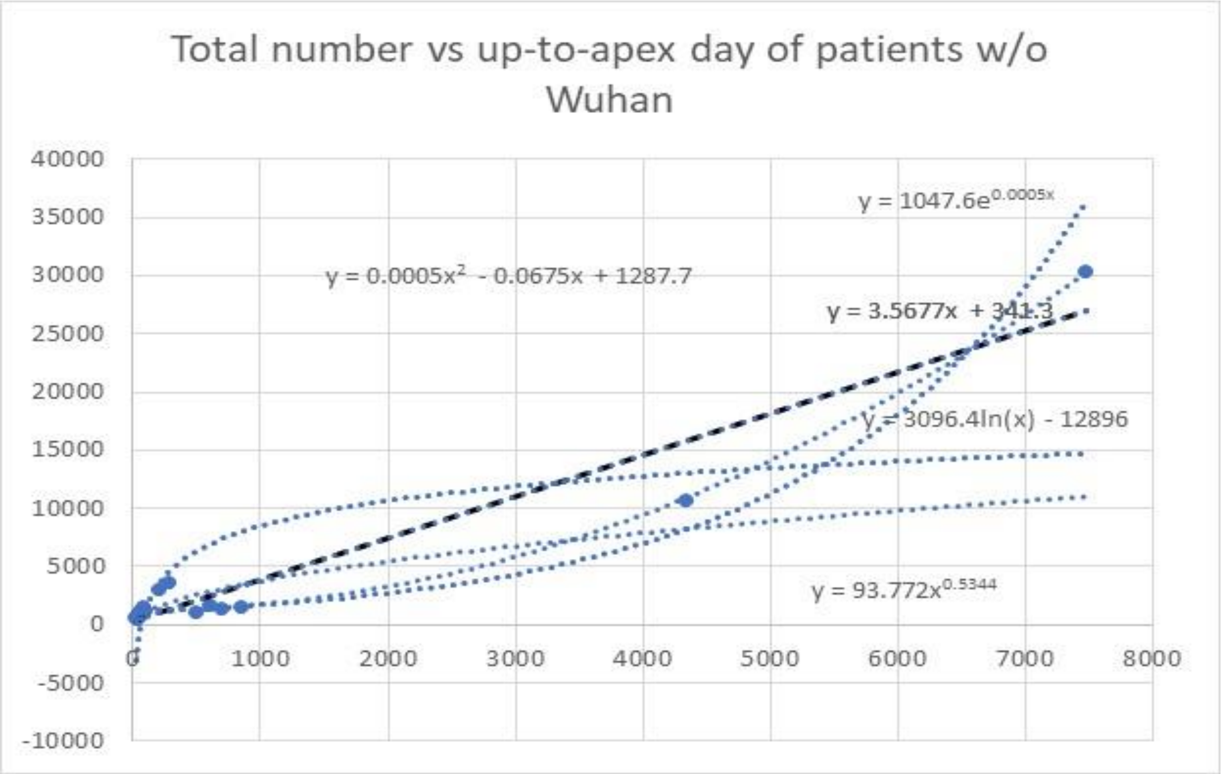

Supplemental Figure 2**C.**

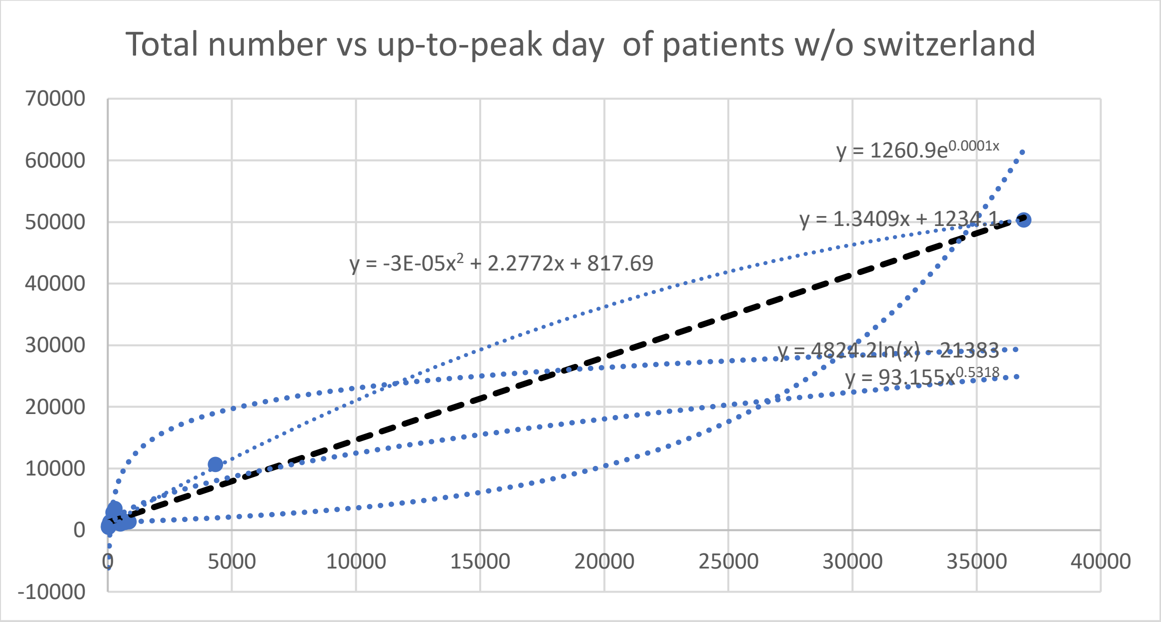

Supplemental Figure 2**D.**

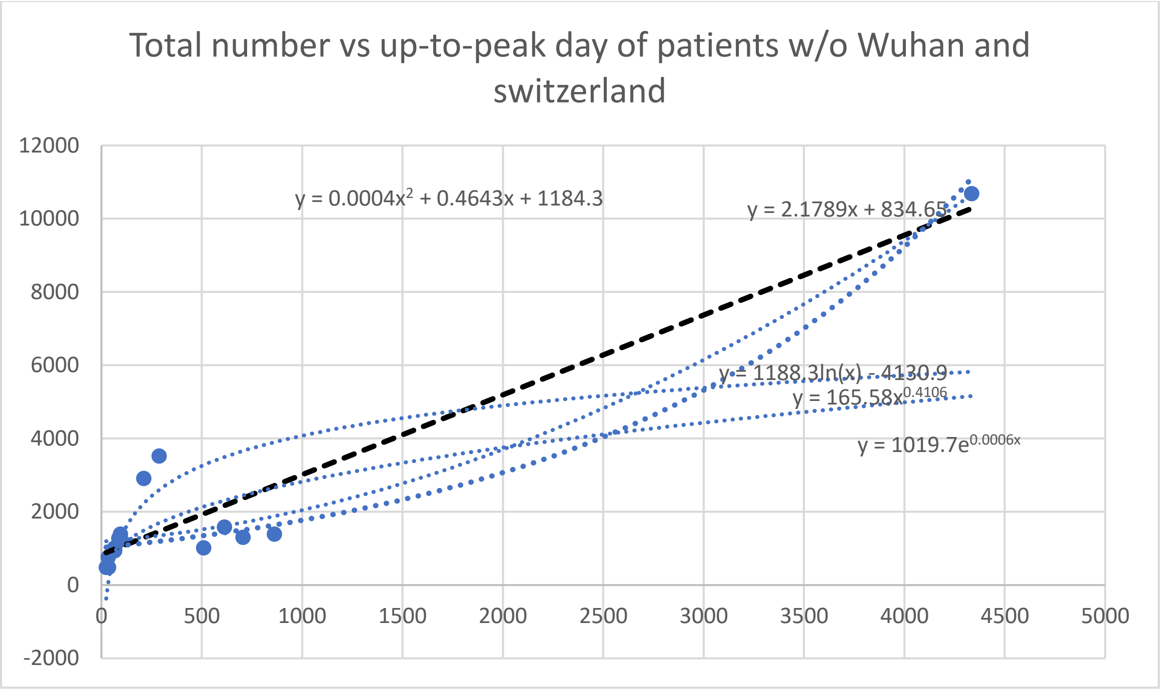

Total number vs up-to-peak day of patients w/o Wuhan and switzerland
12000
10000
8000
6000
4000
2000
0
-2000
0
500
1000
1500
2000
2500
3000
3500
4000
4500
5000
y = 0.0004x2 + 0.4643x + 1184.3
y = 2.1789x + 834.65
y = 1188.3ln(x) - 4130.9
y = 165.58x0.4106
y = 1019.7e0.0006x
