# Supplementary material for: Toward a Country-Based Prediction Model of COVID-19 Infections and Deaths Between Disease Apex and End: Evidence From Countries With Contained Numbers of COVID-19
Source: Front Med (Lausanne). 2021 Jun 10;8:585115. doi: 10.3389/fmed.2021.585115 (PMC8222531; doi:10.3389/fmed.2021.585115)

Supplemental Figure 3. Death numbers and relations before peak day and the total death

Supplemental Figure 3A. death number before apex (peak) day vs total death

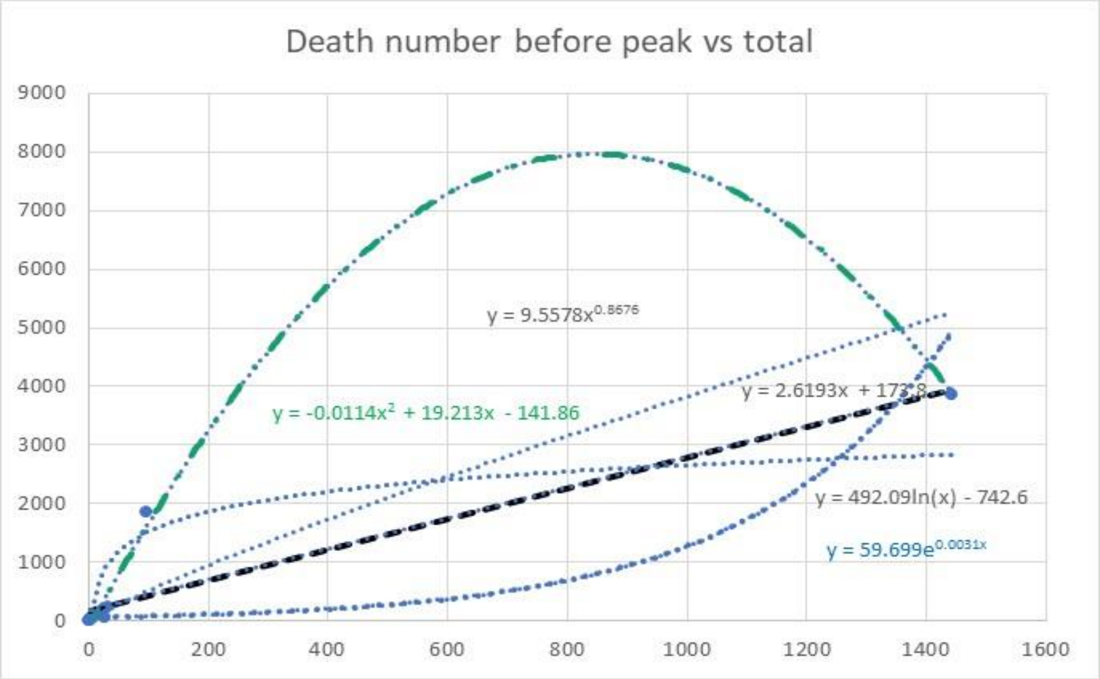

Supplemental Figure 3B. Death numbers and relations before peak day and the total (W/o Wuahn)

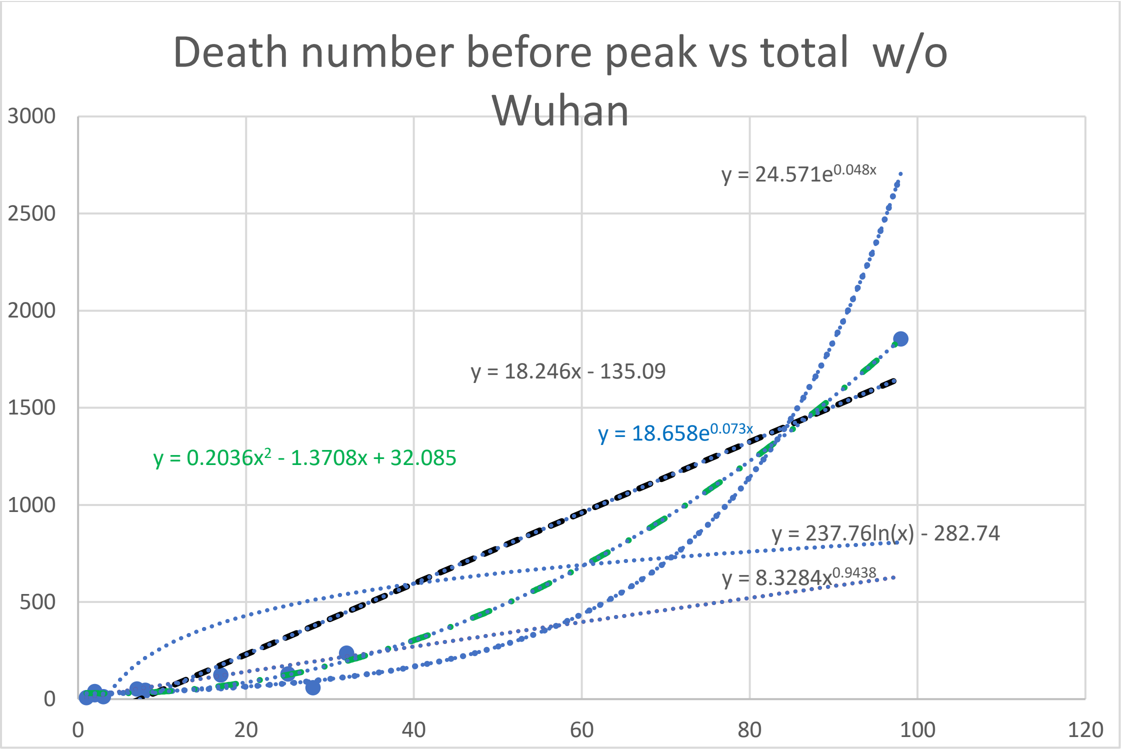

Supplemental Figure 3C. Total death vs death on and before peak day W/O Switzerland

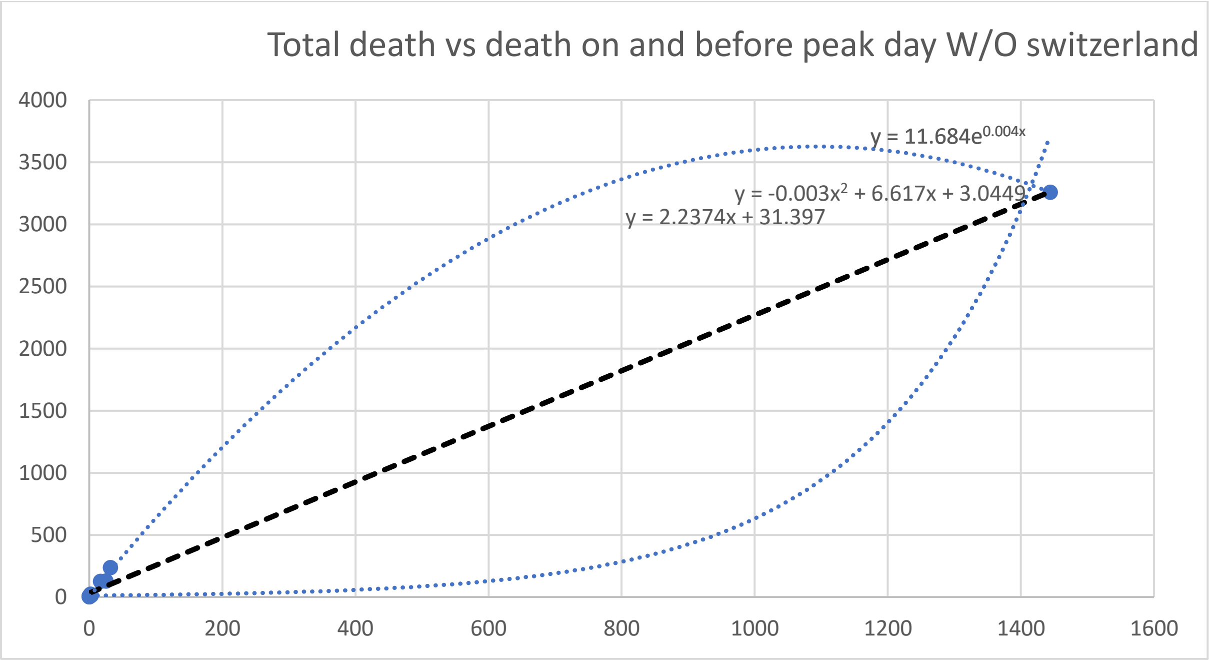

Supplement: Supplementary Figure 3 — Death numbers and relations before apex day and total. [file Data_Sheet_3.PDF]
