# Supplementary material for: Toward a Country-Based Prediction Model of COVID-19 Infections and Deaths Between Disease Apex and End: Evidence From Countries With Contained Numbers of COVID-19
Source: Front Med (Lausanne). 2021 Jun 10;8:585115. doi: 10.3389/fmed.2021.585115 (PMC8222531; doi:10.3389/fmed.2021.585115)

Supplemental Figure 4. Patterns of numbers of infected person and peak day of investigated countries.

Supplemental Figure 4A. Patterns of infected persons and peak day of Japan

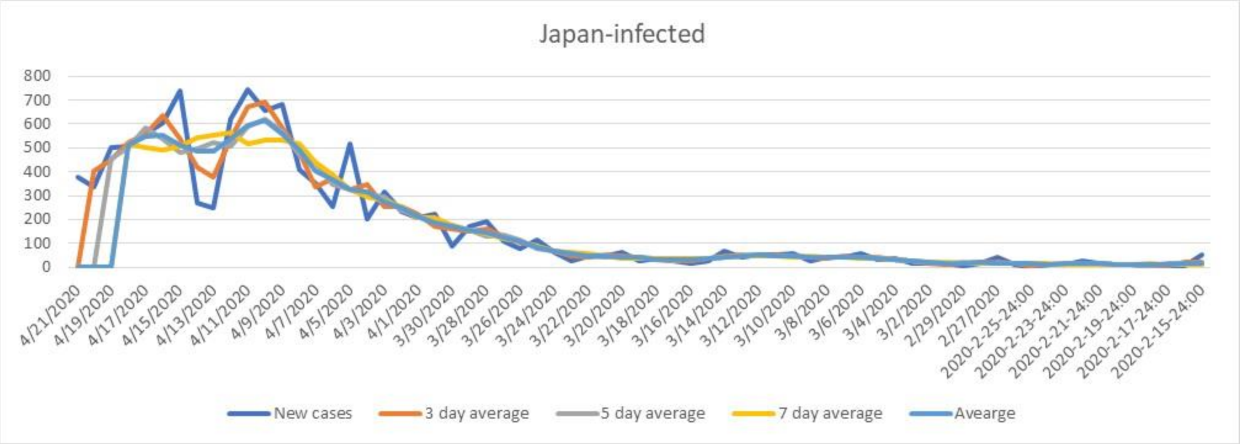

Supplemental Figure 4B. Patterns of infected persons and peak day of Japan

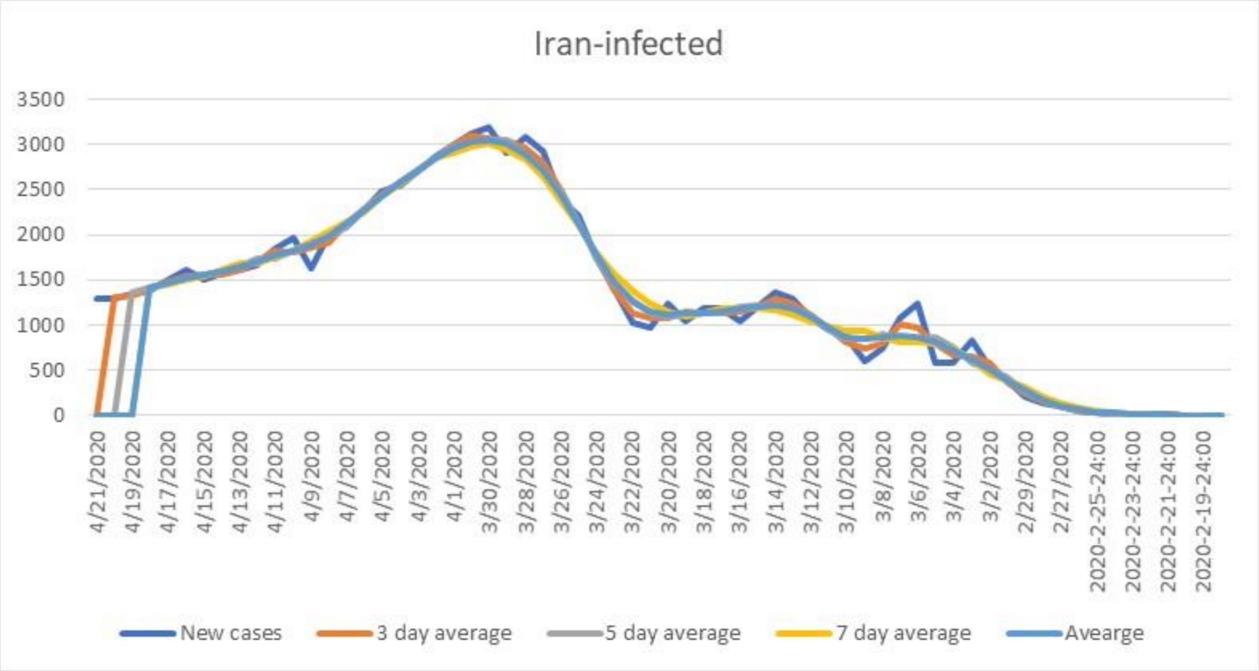

Supplemental Figure 4C. Patterns of infected persons and peak day of France

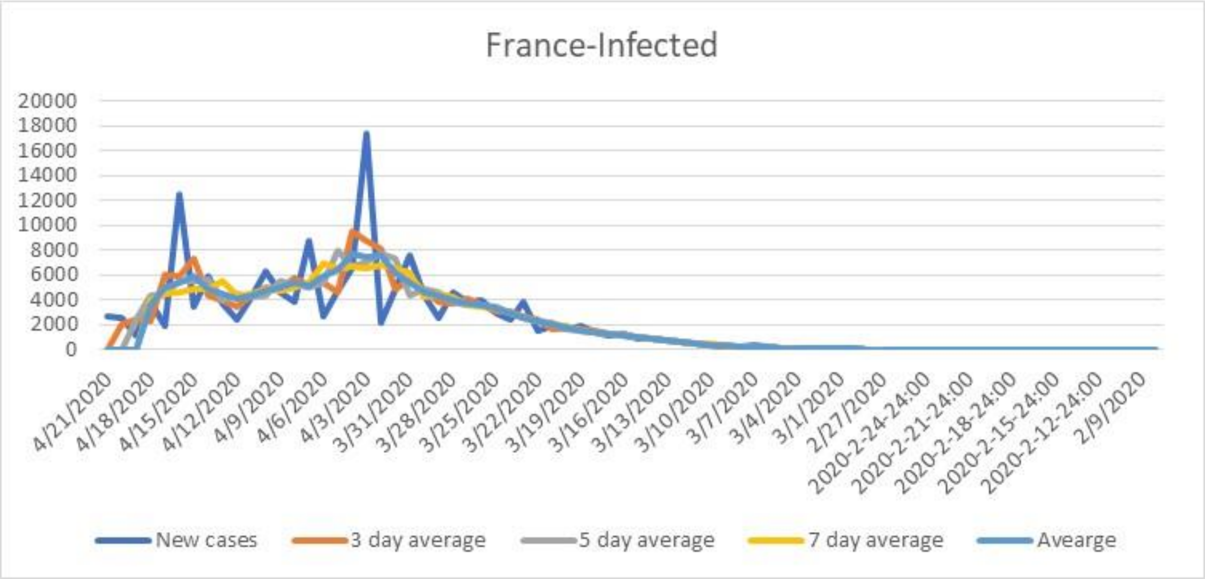

Supplemental Figure 4D. Patterns of infected persons and peak day of Italy

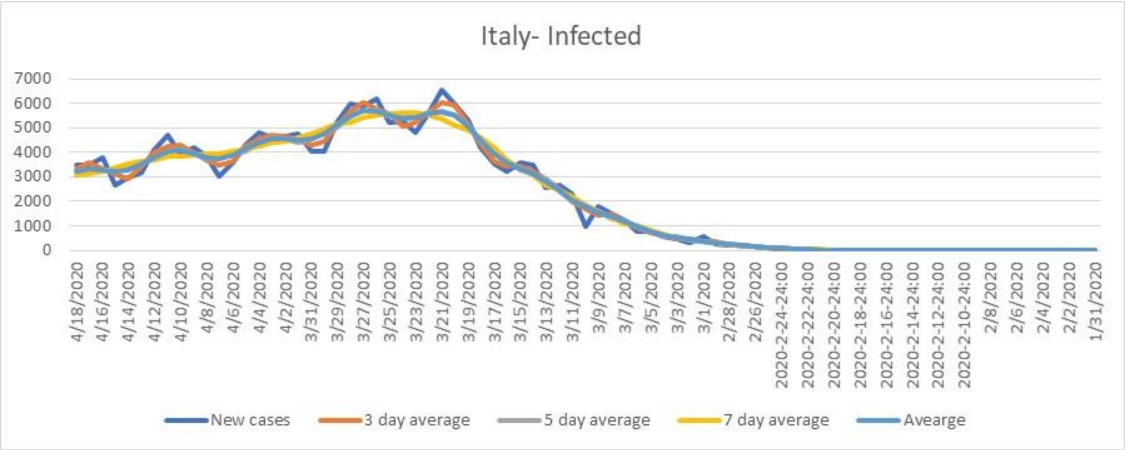

Supplemental Figure 4E. Patterns of infected persons and peak day of Spain

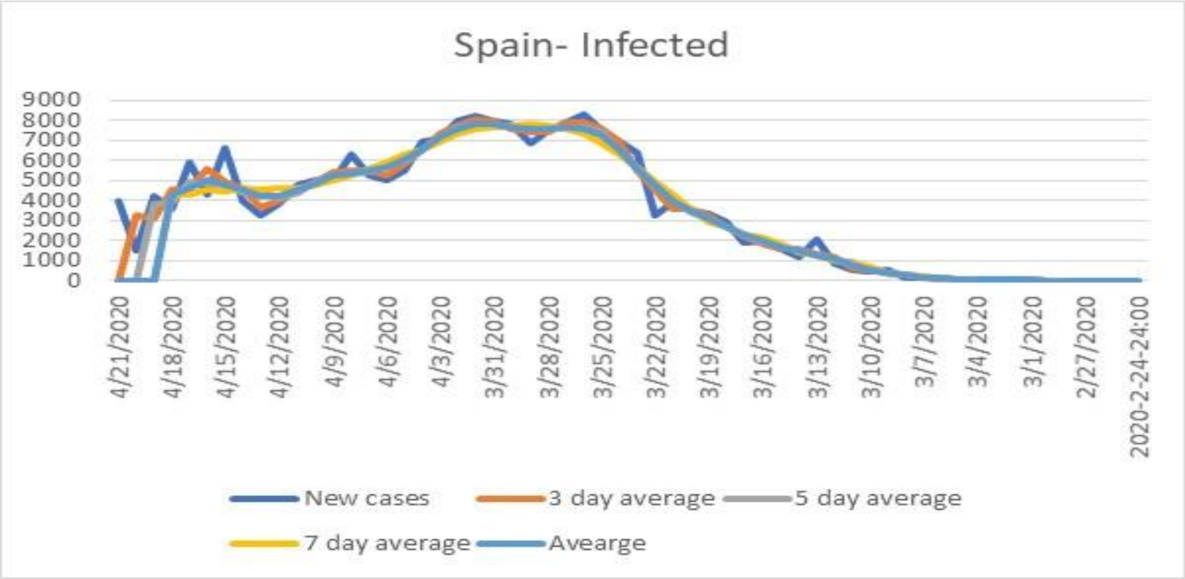

Supplemental Figure 4F. Patterns of infected persons and peak day of Germany

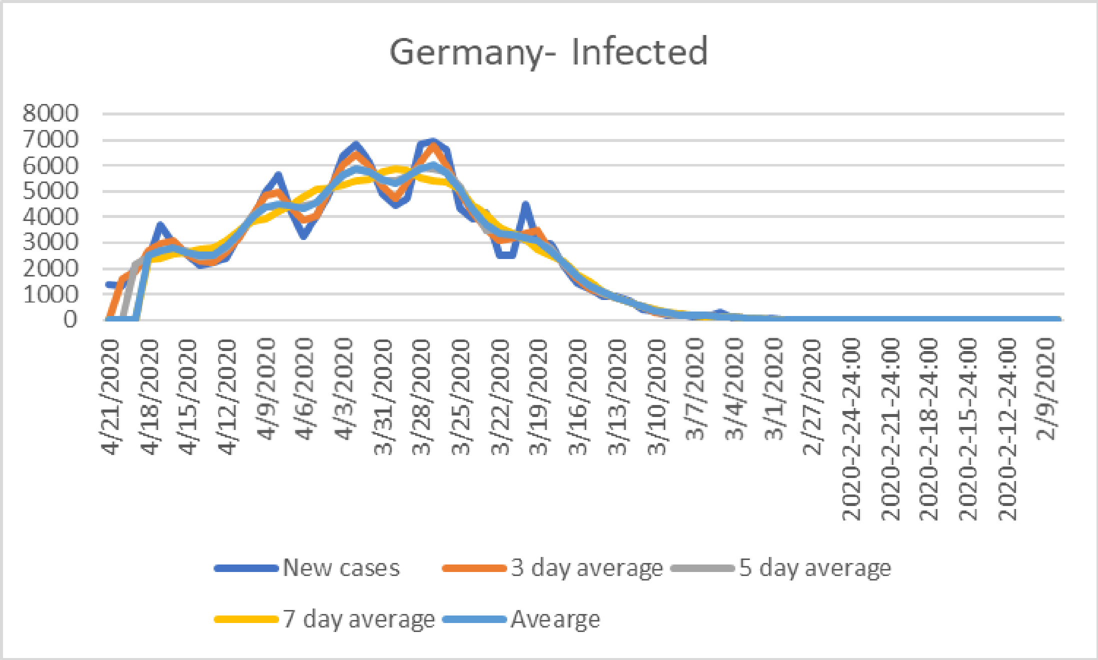

Supplemental Figure 4G. Patterns of infected persons and peak day of UK

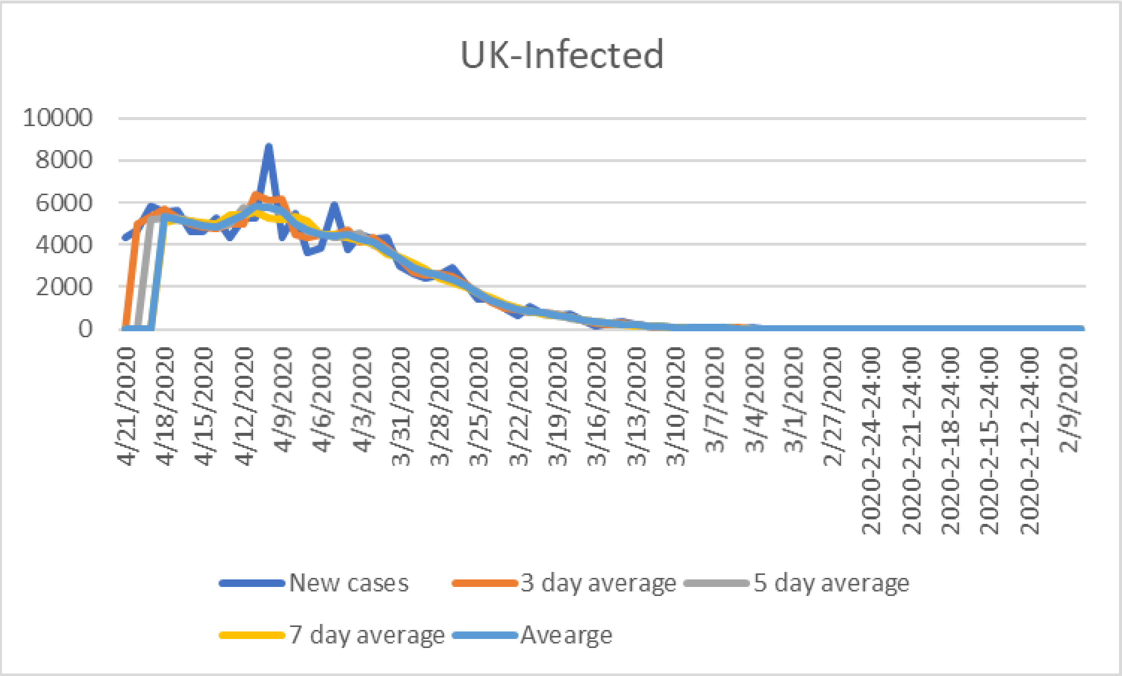

Supplemental Figure 4H. Patterns of infected persons and peak day of Netherland

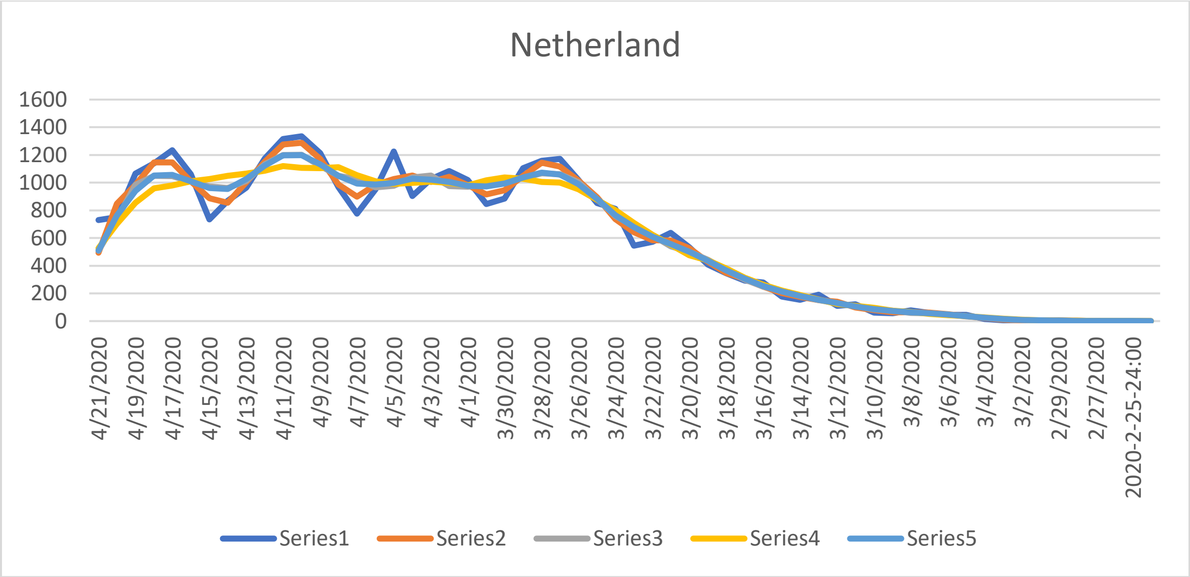

Supplemental Figure 4I. Patterns of infected persons and peak day of Belgium

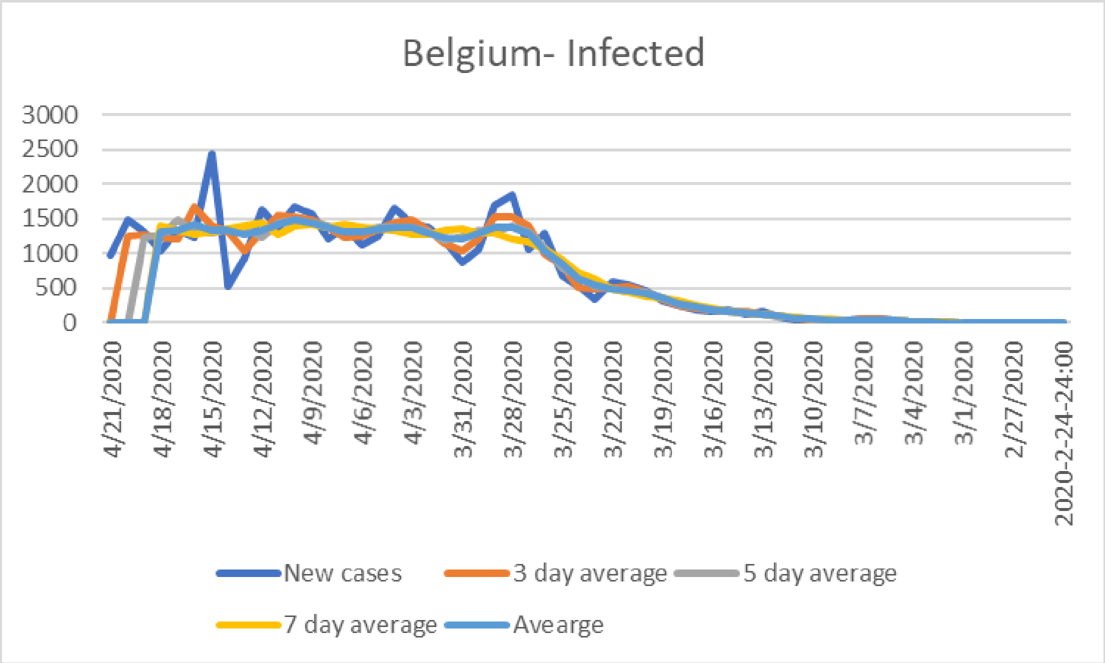

Supplemental Figure 4J. Patterns of infected persons and peak day of US

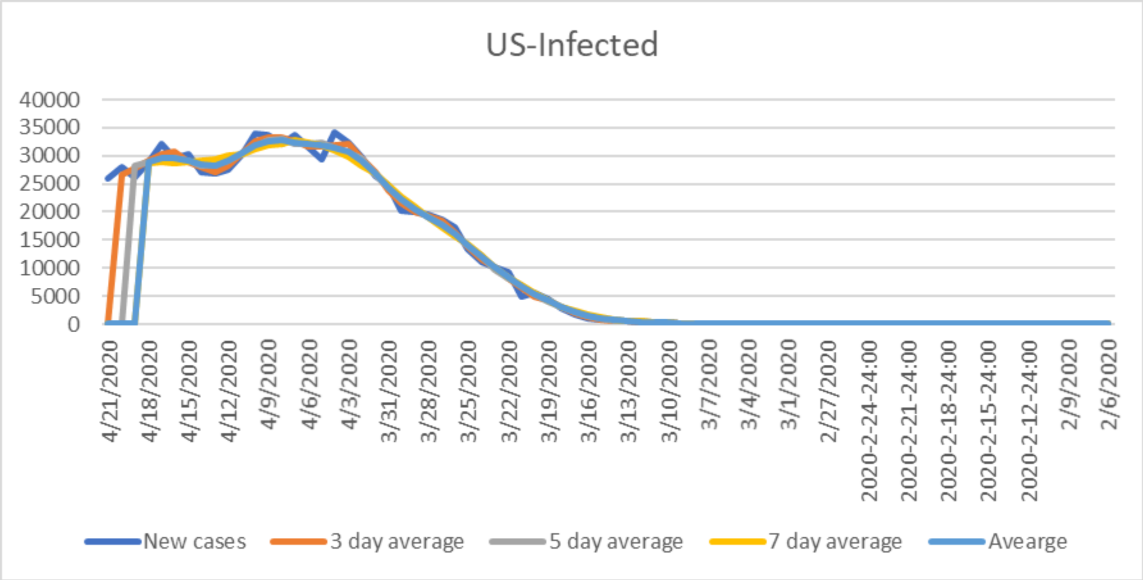

Supplement: Supplementary Figure 4 — Patterns of numbers of infected persons and peak day of investigated countries. [file Data_Sheet_4.PDF]
