# Supplementary material for: Toward a Country-Based Prediction Model of COVID-19 Infections and Deaths Between Disease Apex and End: Evidence From Countries With Contained Numbers of COVID-19
Source: Front Med (Lausanne). 2021 Jun 10;8:585115. doi: 10.3389/fmed.2021.585115 (PMC8222531; doi:10.3389/fmed.2021.585115)
Supplement: Supplementary Table 1 — Days before and after apex day. [file Data_Sheet_5.pdf]

Supplemental Table 1. Calculation of the remaining days after April 21 to the end of the COVID-19 epidemic

Country	Days to apex day	days till 5/10		$y = 1.4402x + 11.524$	remain days	$y = 0.0043x^2 + 1.2886x + 12.716$	remain days
Japan	53	87	34	88	54	93	59
Iran	41	83	42	71	29	73	31
France	68	94	26	109	83	120	94
Italy	57	102	45	94	49	100	55
Spain	62	102	40	101	61	109	69
Germany	49	94	45	82	37	86	41
UK	64	102	38	104	66	113	75
Netherland	44	75	31	75	44	78	47
US	63	96	33	102	69	111	78
Belgium	47	78	31	79	48	83	52
S Korea	17			36		36	

Country	Day to peak	$y = 17.635e^{0.0384x}$	remain days
Japan	67	135	101
Iran	63	85	43
France	74	240	214
Italy	82	157	112
Spain	82	191	151
Germany	74	116	71
UK	82	206	168
Netherland	55	96	65
US	76	198	165
Belgium	58	107	76
