# Supplementary material for: Toward a Country-Based Prediction Model of COVID-19 Infections and Deaths Between Disease Apex and End: Evidence From Countries With Contained Numbers of COVID-19
Source: Front Med (Lausanne). 2021 Jun 10;8:585115. doi: 10.3389/fmed.2021.585115 (PMC8222531; doi:10.3389/fmed.2021.585115)
Supplement: Supplementary Table 2 — Calculation of the numbers of patients from the first wave. [file Data_Sheet_6.pdf]

Supplemental Table 2. Calculation of the numbers of patients during first wave of the COVID-19 disease linear and polynomial models.

Country	Infected till peak	# end of 1 wave	y = 1.4086x + 2097.3	y = 3.5677x + 341.3 W/o Wuh	y = 1.3409x + 1234.1 W/o Swit
Japan 5/31-4/13 2020	7618	16851	12828	27520	11449
Iran 5/16/-4/2 2020	50468	11635	73187	180396	68907
France 6/16/ - 4/1/2020	56989	156156	82372	203661	77651
Italy 6/5/-3/25/2020	74386	234013	106877	265728	100978
Spain 6/12/- 3/21/2020	25496	242707	38011	91303	35422
Germany 6/11/- 3/30/2020	66885	185416	96312	238967	90920
UK 7/6/-4/13/2020	88621	285420	126929	316514	120066
Netherland 6/27/- 4/13/2020	26551	50005	39497	95067	36836
Belgium 6/23/- 4/13/2020	30589	60550	45185	109474	42251
US 5/24/-4/10/2020	505959	1568448	714791	1805451	679675
Brazil 11/8- 7/26/2020	2343366	5631181	3302963	8360768	3143454
India 2/9/2021- 9/13/2020	4754356	10826363	6699083	16962457	6376350
Russia 8/23/- 5/12/2020-	232243	956749	329235	828915	312649
Turkey 6/13/- 4/14/2020	61049	175218	88091	218146	83095

Poly	# end of 1 wave	$y = -7E-05x^2 + 4.011x + 254.44$	$y = 0.0005x^2 - 0.0675x + 1287.7$ W/o Wuh	$y = -3E-05x^2 + 2.2772x + 817.69$ W/O swit
Japan	16851	30810.238	29790.447	-10851.5624
Iran	11635	202681.588	1271390.622	-1157766.09
France	156156	228837.319	1621314.003	-1493280.02
Italy	234013	298616.686	2762905.143	-2596429.01

Spain	242707	102518.896	324589.728	-266145.827
Germany	185416	268530.175	2233574.575	-2083673.4
UK	285420	355713.271	3922146.603	-3724215.39
Netherland	50005	106750.501	351973.308	-291198.173
Belgium	60550	122946.919	467066.403	-397368.5
US	1568448	2029655.989	127964390.3	-126844267
Brazil	5631181	9399495.466	2745525215	-2740344974
India	10826363	19069976.36	11301630856	-1.1291E+10
Russia	956749	931781.113	26954016.82	-26438724.1
Turkey	175218	245121.979	1860657.093	-1723651.73
