# Supplementary material for: Toward a Country-Based Prediction Model of COVID-19 Infections and Deaths Between Disease Apex and End: Evidence From Countries With Contained Numbers of COVID-19
Source: Front Med (Lausanne). 2021 Jun 10;8:585115. doi: 10.3389/fmed.2021.585115 (PMC8222531; doi:10.3389/fmed.2021.585115)
Supplement: Supplementary Table 3 — Calculation of the numbers of deaths from the first wave. [file Data_Sheet_7.pdf]

Supplemental Table 3. Calculation of the numbers of deaths first during wave of the COVID-19 disease linear and polynomial models.

Country	Death till Peak	# end of 1 wave	y = 2.6193x + 173.8	y = 18.246x - 135.09 w/o Wuh	**y = 2.6534x + 39.527 w/o switz**
Japan 5/31-4/13 2020	143	891	548	**2474**	419
Iran 5/16/-4/2 2020	3160	6902	8451	**57522**	8424
France 6/16/ - 4/1/2020	4032	29700	10735	**73433**	10738
Italy 6/5/-3/25/2020	7503	33689	19826	**136765**	19948
Spain 6/12/- 3/21/2020	1381	27136	3791	**25063**	3704
Germany 6/11/- 3/30/2020	645	8755	1863	**11634**	1751
UK 7/6/-4/13/2020	11329	44220	29848	**206574**	30100
Netherland 6/27/- 4/13/2020	964	6103	2699	**17454**	2597
Belgium 6/23/- 4/13/2020	3903	9696	10397	**71079**	10396
US 5/24/-4/10/2020	21830	94011	57353	**398175**	57963
Brazil 11/8- 7/26/2020	85238	162015	223438	**1555117**	226210
India 2/9/2021- 9/13/2020	78586	154996	206014	**1433745**	208560
Russia 8/23/- 5/12/2020-	2116	16383	5716	**38473**	5654
Turkey 6/13/- 4/14/2020	1296	4778	3568	**23512**	3478
			40	11	

Poly	# end of 1 wave	**$y = -0.0114x^2 + 19.213x - 141.86$**	**$y = 0.2036x^2 - 1.3708x + 32.085$ W/o Wuh**	**$y = -0.0016x^2 + 4.9815x + 11.088$ W/o Swit**
Japan	891	2372.4804	3999.477	690.7241
Iran	6902	-53264.62	2028768.517	-224.332
France	29700	-108005.1176	3304435.106	-5914.7424
Italy	33689	-497749.8236	11451410.81	-52684.7319
Spain	27136	4649.6576	386436.9898	3839.0819
Germany	8755	7507.84	83850.609	2558.5155
UK	44220	-1245624.93	26115796.96	-148907.4841
Netherland	6103	7785.4976	187915.2994	3326.3804
Belgium	9696	-98814.3836	3096203.925	-4919.5719

US	94011	-5013379.53	96995463.56	-653721.007
Brazil	162015	-81189353.91	1479142377	-11200202.45
India	154996	-68893926.16	1257276919	-9489727.787
Russia	16383	-10530.1504	908741.5138	3388.0124
Turkey	4778	5610.5656	340225.3458	3779.7264
