# Supplementary material for: Toward a Country-Based Prediction Model of COVID-19 Infections and Deaths Between Disease Apex and End: Evidence From Countries With Contained Numbers of COVID-19
Source: Front Med (Lausanne). 2021 Jun 10;8:585115. doi: 10.3389/fmed.2021.585115 (PMC8222531; doi:10.3389/fmed.2021.585115)
Supplement: Supplementary Table 4 — Calculation of the numbers of patients from the entire pandemic. [file Data_Sheet_8.pdf]

Supplemental Table 4. Calculation of the numbers of patients update to March 30, 2021 of the COVID-19 disease linear and polynomial models.

Country	Infected till peak	3/30/2021	y = 1.4086x + 2097.3	y = 3.5677x + 341.3 W/o Wuh	y = 1.3409x + 1234.1 W/o Swit
Japan 1/10/2021	280775	466849	397597	1002062	377725
Iran 12/1/2020	935799	1846923	1320264	3338991	1256047
France Nov 10 2020	1714361	4435057	2416946	6116667	2300021
Italy 11/17/2020	1144552	3512453	1614313	4083759	1535964
Spain 1/10/2021	2025560	3247738	2855301	7226932	2717308
Germany 12/20/2020	1494009	2772401	2106558	5330517	2004551
UK 1/10/2021	3017413	4329184	4252425	10765566	4047283
Netherland 12/20/2020	676589	1244924	955141	2414208	908472
Belgium 11/1/2020	445795	870757	630044	1590804	599001
US 1/10/2021	21761186	29859706	30654904	77637725	29180808
Brazil july 26 2020	2343366	12404414	3302963	8360768	3143454
India 9/13 2020	4754356	11971624	6699083	16962457	6376350
Russia 12/27/2020	3050248	4519832	4298677	10882711	4091312
Turkey 12/13/2020	995471	3179115	1404318	3551883	1336061

Poly	3/30/2021	Future Patient 1	Future Patient 2	Future Patient 3
Japan	466849	-69252.035	535213	-89123.7
Iran	1846923	-526659.2286	1492068	-590876.0
France	4435057	-2018110.795	1681610	-2135036.2
Italy	3512453	-1898139.753	571306	-1976489.1
Spain	3247738	-392436.884	3979194	-530430.5
Germany	2772401	-665842.6226	2558116.209	-767850.2
UK	4329184	-76758.7482	6436381.66	-281900.8
Netherland	1244924	-289783.4346	1169283.875	-336451.7
Belgium	870757	-240712.863	720047.1215	-271756.4

US	29859706	795197.8996	47778019	-678897.6
Brazil	12404414	-9101451.352	-4043646	-9260960.4
India	11971624	-5272540.838	4990833	-5595273.9
Russia	4519832	-221155.3672	6362879	-428520.4
Turkey	3179115	-1774797.249	372768	-1843053.8
