# Supplementary material for: Toward a Country-Based Prediction Model of COVID-19 Infections and Deaths Between Disease Apex and End: Evidence From Countries With Contained Numbers of COVID-19
Source: Front Med (Lausanne). 2021 Jun 10;8:585115. doi: 10.3389/fmed.2021.585115 (PMC8222531; doi:10.3389/fmed.2021.585115)
Supplement: Supplementary Table 5 — Calculation of the numbers of deaths from the entire pandemic. [file Data_Sheet_9.pdf]

Supplemental Table 5. Calculation of the numbers of deaths update to March 30, 2021 of the COVID-19 disease linear and polynomial models.

Country	Death till Peak	3/30/2021	y = 2.6193x + 173.8	y = 18.246x - 135.09 w/o Wuh	**y = 2.6534x + 39.527 w/o switz**
Japan 1/10/2021	3996	9031	10641	**72776**	10643
Iran 12/1/2020	47486	62308	124554	**866294**	126039
France 11/10/2020	39849	93884	104550	**726950**	105775
Italy 11/17/2020	44683	107636	117212	**815151**	118601
Spain 1/10/2021	51690	74420	135565	**943001**	137194
Germany 12/20/2020	26049	75870	68404	**475155**	69158
UK 1/10/2021	80868	126573	211991	**1475382**	214615
Netherland 12/20/2020	10454	16450	27556	**190609**	27778
Belgium 11/1/2020	11734	22897	30909	**213963**	31175
US 1/10/2021	365886	543003	958539	**6675821**	970881
Brazil 7/26/2020	85238	307112	223438	**1555117**	226210
India 11/13,2020	78586	161552	206014	**1433745**	208560
Russia 12/27/2020	54778	97740	143654	**999344**	145387
Turkey 12/13/2020	16199	30923	42604	**295432**	43022

Poly	3/30/2021	future death	W/o Wuh	W/0 Swi	W/o Wuh Swit
Japan	9031	1609.5228	63744.926	1611.5134	10694.0994
Iran	62308	62245.8798	803986.466	63730.8794	171970.6654
France	93884	10666.2857	633065.764	11890.8636	102718.2896
Italy	107636	9575.9819	707514.928	10965.3992	112814.3452
Spain	74420	61145.417	868580.65	62773.773	180598.679
Germany	75870	-7466.0543	399284.964	-6712.0564	52651.3696
UK	126573	85418.3524	1348809.438	88041.6782	272392.4242
Netherland	16450	11105.9622	174158.594	11328.1706	35134.9966
Belgium	22897	8011.6662	191066.474	8277.5226	35002.7486
US	543003	415535.9998	6132817.866	427878.4394	1262070.225
Brazil	307112	-83674.3066	1248005.458	-80901.9638	113412.3822
India	161552	44462.1098	1272193.066	47007.6194	226155.4054
Russia	97740	45913.8154	901604.298	47647.4722	172513.0182
Turkey	30923	11680.8407	264508.864	12098.9536	49004.3796
